# The efficiency of convalescent plasma in COVID-19 patients: A systematic review and meta-analysis of randomized controlled clinical trials

**DOI:** 10.3389/fimmu.2022.964398

**Published:** 2022-07-28

**Authors:** Zhenbei Qian, Zhijin Zhang, Haomiao Ma, Shuai Shao, Hanyujie Kang, Zhaohui Tong

**Affiliations:** Department of Respiratory and Critical Care Medicine, Beijing Institute of Respiratory Medicine, Beijing Chao-Yang Hospital, Capital Medical University, Beijing, China

**Keywords:** convalescent plasma, COVID-19, SARS-CoV-2, antibodies, mortality, passive immunization

## Abstract

The objective of this study was to assess whether convalescent plasma therapy could offer survival advantages for patients with novel coronavirus disease 2019 (COVID-19). An electronic search of Pubmed, Web of Science, Embase, Cochrane library and MedRxiv was performed from January 1st, 2020 to April 1st, 2022. We included studies containing patients with COVID-19 and treated with CCP. Data were independently extracted by two reviewers and synthesized with a random-effect analysis model. The primary outcome was 28-d mortality. Secondary outcomes included length of hospital stay, ventilation-free days, 14-d mortality, improvements of symptoms, progression of diseases and requirements of mechanical ventilation. Safety outcomes included the incidence of all adverse events (AEs) and serious adverse events (SAEs). The Cochrane risk-of-bias assessment tool 2.0 was used to assess the potential risk of bias in eligible studies. The heterogeneity of results was assessed by I^2 test and Q statistic test. The possibility of publication bias was assessed by conducting Begg and Egger test. GRADE (Grading of Recommendations Assessment, Development and Evaluation) method were used for quality of evidence. This study had been registered on PROSPERO, CRD42021273608. 32 RCTs comprising 21478 patients with Covid-19 were included. Compared to the control group, COVID-19 patients receiving CCP were not associated with significantly reduced 28-d mortality (CCP 20.0% vs control 20.8%; risk ratio 0.94; 95% CI 0.87-1.02; p = 0.16; I² = 8%). For all secondary outcomes, there were no significant differences between CCP group and control group. The incidence of AEs (26.9% vs 19.4%,; risk ratio 1.14; 95% CI 0.99-01.31; p = 0.06; I² = 38%) and SAEs (16.3% vs 13.5%; risk ratio 1.03; 95% CI 0.87-1.20; p = 0.76; I² = 42%) tended to be higher in the CCP group compared to the control group, while the differences did not reach statistical significance. In all, CCP therapy was not related to significantly improved 28-d mortality or symptoms recovery, and should not be viewed as a routine treatment for COVID-19 patients.

**Trial registration number:**

CRD42021273608. Registration on February 28, 2022

**Systematic review registration:**

https://www.crd.york.ac.uk/prospero/, Identifier CRD42022313265.

## 1 Introduction

The coronavirus disease 2019 (COVID-19), which was caused by the infection of Severe Acute Respiratory Syndrome Coronavirus 2 (SARS-COV-2), had become an acknowledged global pandemic and accounted for more than five hundred million confirmed cases and six million deaths (1), bringing a heavy burden to the healthcare system and serious threat to human beings. At present, the majority of treatments were still supportive, while few therapeutic strategies were confirmed for improved survival benefits.

Convalescent Plasma (CP) therapy, a form of passive immunization, had been widely applied in many viral infectious diseases like Middle East respiratory syndrome (MERS) (2) and Ebola (3). The specific antibodies in CP could accelerate clearance of virus (4), promote antibody-dependent cell-mediated cytotoxicity and complement activation (5). Results from previous studies suggested reduced mortality and improved symptoms in COVID-19 patients treated with COVID-19 convalescent plasma (CCP) (6, 7), and the FDA of the United States had approved the emergency use authorization (EUA) of CCP in COVID-19 patients (8). However, these studies were mainly retrospective and contained potential risk of bias, while the results from prospective studies suggested that administration of CCP could not result in reduced risk of mortality or improved symptoms (9–11). Recent meta-analysis which included results from RCTs (8, 12, 13) also indicated no significant improvements in the survival of COVID-19 who received CCP. The inconsistence of these studies made it controversial whether CCP should be regarded as a routine therapy for COVID-19 patients.

To further assess the efficiency of CCP, we conducted this meta-analysis to systematically evaluate whether COVID-19 patients could benefit from CCP therapy.

## 2 Methods

We reported this study according to the Preferred Reporting Items for Systematic Reviews and Meta-analyses (PRISMA) guidelines (14). We have registered this study on PROSPERO (CRD42022313265) on February 28, 2022.

### 2.1 Inclusion and exclusion criteria

Studies were included if they fulfilled the following inclusion criteria: 1) Patients were confirmed at any clinical stage of COVID-19. 2) Patients ≥18 years old. 3) The intervention should be convalescent plasma. 4) The control group should include contemporaneous patients who didn’t receive CCP or were treated with a placebo, including normal saline or standard plasma. 5) Only randomized controlled clinical trials were included. Exclusion criteria were defined as followed: 1) Animal or cell studies. 2) Editors, reviews, comments or abstracts. 3) Studies with unavailable full text. 4) Ineligible study designs, e.g. observational studies, retrospective studies, case reports, or case series studies. 5) Studies only contain the results that we were not interested in, including the changes in inflammatory factors (e.g. ferritin, IL-10 and D-Dimmer) or biochemical factors (e.g. bilirubin, albumin and creatinine), the proportion of patients with negative nucleic acid test, time to the negative nucleic acid test, the proportion of patients with detectable endogenous antibodies after receiving CCP.

### 2.2 Search strategy

We performed a comprehensive search of the database including Pubmed, Web of Science, Embase, Cochrane library and medRxiv from January 1st, 2020 to April 1st, 2022. The keywords of “COVID-19” and “convalescent plasma” were used. No language restrictions were applied. Detailed systematic search strategy could be found in **Additional Table 1**. Reference lists of eligible studies were manually screened in case of loss of potentially relevant publications. The identification of potentially eligible studies was independently performed by two reviews (ZB Qian and S Shao). Any disagreement or discrepancy was eventually resolved by a third reviewer (ZH Tong).

### 2.3 Data collection and quality assessment

Two reviews (ZJ Zhang and HM Ma) conducted data collection independently. Any disagreement was resolved by the third reviewer (ZH Tong). For candidate literature, we designed a data collection form for temporary data management. The following information was extracted: name of the first author, publication year, study design, registration ID, inclusion criteria of subjects, the titer of neutralizing antibody and the dosage of CCP, type of control, sample size, details of baseline conditions and clinical outcomes. The incomplete data would be estimated by estimation or obtained by contacting the corresponding author. The Cochrane risk-of-bias assessment tool 2.0 (RoB 2.0) (15) was used to examine the potential risk of bias in eligible studies.

### 2.4 Outcomes

The selection and definition of outcomes referred to the previous meta-analysis (8, 12) and RCTs. The primary outcome was the 28-d mortality. Key secondary outcomes included 14-d mortality, the length of hospital stay (LOS), ventilation-free days, improvements of symptoms, progression of diseases and requirement of mechanical ventilation. The LOS was defined as the time from admission to hospital to discharge or death. Ventilation-free days were defined as the days without the support of ventilation. LOS and ventilation-free days were both assessed on day 28 (16). Improvements of symptoms were defined as improvements at least 2 grades on the WHO 7 symptom score within 28 days (17), while the progression of disease was defined as an exacerbation of the WHO 7 symptom score for at least 2 points or requirement for invasive ventilation or death. The safety outcomes included the incidence of all adverse events and serious adverse events. Severe adverse events referred to the adverse events that were assessed grade 3 or 4 (18).

### 2.5 Data synthesis

For continuous variables including LOS and ventilation-free days, the mean and standard deviation (SD) were extracted to calculate the mean difference (MD) with a 95% confidence interval (95% CI). For categorical variables like mortality, improvements of symptoms, progression of diseases, requirements of MV and incidence of AE, the risk ratio (RR) with 95% CI was calculated from frequencies and percentages. The statistical method was the inverse-variance method for continuous variables, while the Mantel-Haenszel method for categorical variables. All synthesis was based on the random-effects model and a two-tailed value of P less than 0.05 was considered statistically significant for all outcomes. I^2 test and Q statistic test were performed to assess the inter-study heterogeneity, which was defined as moderate-to-high when P<0.1 in Q test and I^2>50%. The possibility of publication bias was assessed by conducting a funnel plot and Egger or Begg test if more than 10 studies were included in the result, which was defined as high when the P value was lower than 0.1. The certainty of the evidence was assessed with the Grading of Recommendations Assessment, Development, and Evaluation tool (GRADE) Profiler version 3.6. Data synthesis was performed by using Review Manager Version 5.4 and Stata software (Stata Statistical Software, release 9.2)

### 2.6 Subgroup analysis and sensitivity analysis

In subgroup analysis, we stratified the eligible studies by (1) The status of publication (published in peer-reviewed publications or at preprint); (2) Patients’ type (outpatients or inpatients); (3) The status of supplementary oxygenation at enrollment (requiring mechanical ventilation(MV), requiring non-invasive ventilation or not requiring oxygenation); (4) The serology of antibody at enrollment (antibody positive or antibody negative); (5) The titer of CCP (high titer CCP, low titer CCP or undivided titer of CCP): In terms of titer determination, we referred to the previous studies [12, 13]. The high titer CCP was defined as long as any of the followings was achieved: a. the titer of S-protein receptor blinding domain specific antibody was more than 1:640; b. the titer of neutralizing antibody was more than 1:40; c. the PRNT50 of anti-S protein specific antibody was more than 1: 320; d. the ID50 of anti-S protein specific antibody was more than 1: 320; e. the signal-to-cutoff (S/C) value of anti-S protein specific antibody was more than 12 (6). The time from symptoms onset to enrollment (no more than 7 days or more than 7 days). The differences across subgroups were considered statistically significant when the P value of the interaction test was lower than 0.05. Forest plots were prepared to graphically visualize the heterogeneity and differences among subgroups. Sensitive analysis was conducted by screening the included studies to assess the impact on the outcomes when I2 ≥50%.

## 3 Results

### 3.1 Literature search and study characteristics

**Figure 1:**
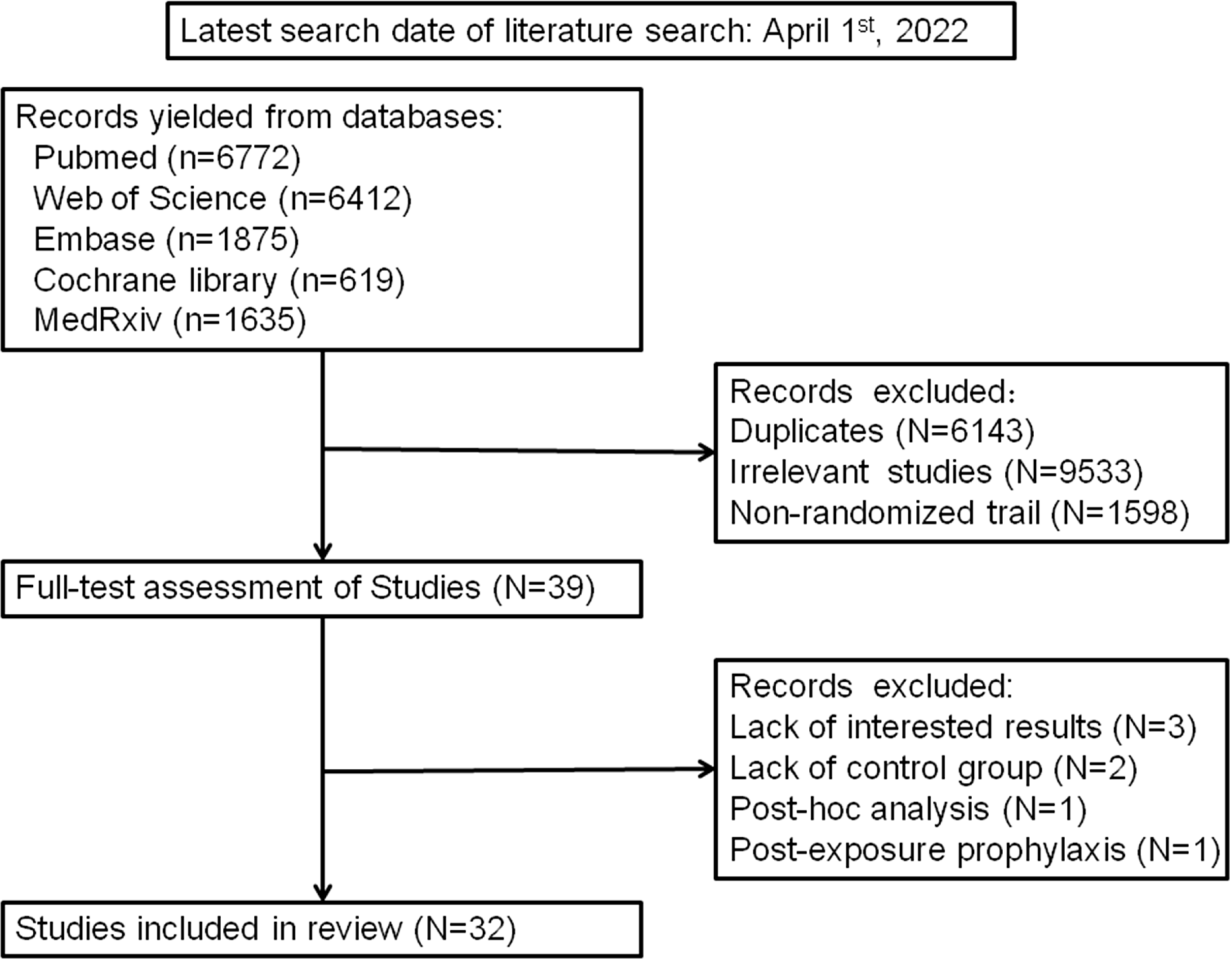
The detailed flow chart of Literature search.

The literature search yielded 17313 records in total, among which 6143 were excluded for duplicates. After the removal of 9533 and 1598 records for irrelevant studies and non-randomized trials, 39 articles were eligible for full-text assessment. Of these, 7 articles were respectively excluded for lack of the results that we were interested in (n=3) including 28-d mortality, changes in the progression of diseases and incidence of adverse events (19–21), lack of control group (n=2) (22, 23), *post-hoc* analysis (n=1) (9) and post-exposure prophylaxis (n=1) (24). Finally, 32 RCTs (16–18, 24–52) with a total of 21478 patients were included in our analysis. A detailed flow chart was shown in **Figure 1**.

### 3.2 Baseline conditions

Among all the included studies, 4 studies (24, 28, 34, 48) were preprinted and 28 studies (16–18, 25–27, 29–33, 35–47, 49–52) were published in peer-reviewed journals. 10 studies (24, 26, 31, 38, 41, 43, 44, 46, 50, 51) were double-blind RCTs with placebo and 22 studies (16–18, 25, 27–30, 32–37, 39, 40, 42, 45, 47–49, 52) were designed as open-label trials. All trials included patients with confirmed Covid-19 except for the RECOVERY trial (35), which included both suspected and confirmed COVID-19 patients. 28 studies focused on the hospitalized patients with supplementary oxygenation (16–18, 25, 27–37, 39, 40, 42–52), and only 4 studies (24, 26, 38, 41) included outpatients. Most patients were older than 60 years and more than 60% were male. The median injection dose of convalescent plasma was 500ml (IQR 250-550). The majority of patients were enrolled more than 7 days after symptom onset. Serum status at enrollment was reported in 14 studies, while the percentage of patients with detectable neutralizing antibodies varied from 11.4% to 83.1% across eligible studies. More detailed information was shown in **Additional Table 2**.

**Figure 2:**
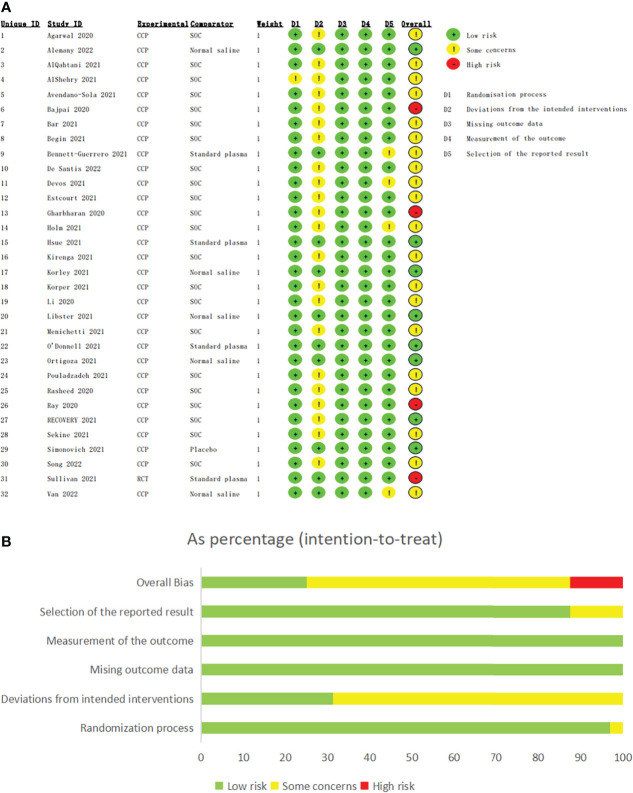
The assessments of risk of bias of eligible studies. The assessments of risk of bias of eligible studies. **(A)** The assessment of each eligible study. **(B)** The assessment of overall bias. Bajpai 2020, Gharbharan 2020, Ray 2020 and Sullivan 2021 were classified as high risk for pre-printed and lack of peer review although no high risks of bias in D1-D5.

The assessments of risk of bias were shown in **Figure 2**. 8 studies (26, 35, 38, 41, 43, 44, 46, 50) were regarded as low risk of bias, and 16 studies (16–18, 25, 27, 29, 30, 32, 37, 39, 40, 42, 45, 47, 49, 52) contained potential performance bias for the open-label design. 4 studies were considered as containing potential bias due to early termination (31, 33, 36, 51). Notably, although no high risks of bias in D1-D5, 4 studies (24, 28, 34, 48) were classified as high risk for pre-printed and lack of peer review.

### 3.3 Synthesis of results

#### 3.3.1 Primary outcome

**Figure 3:**
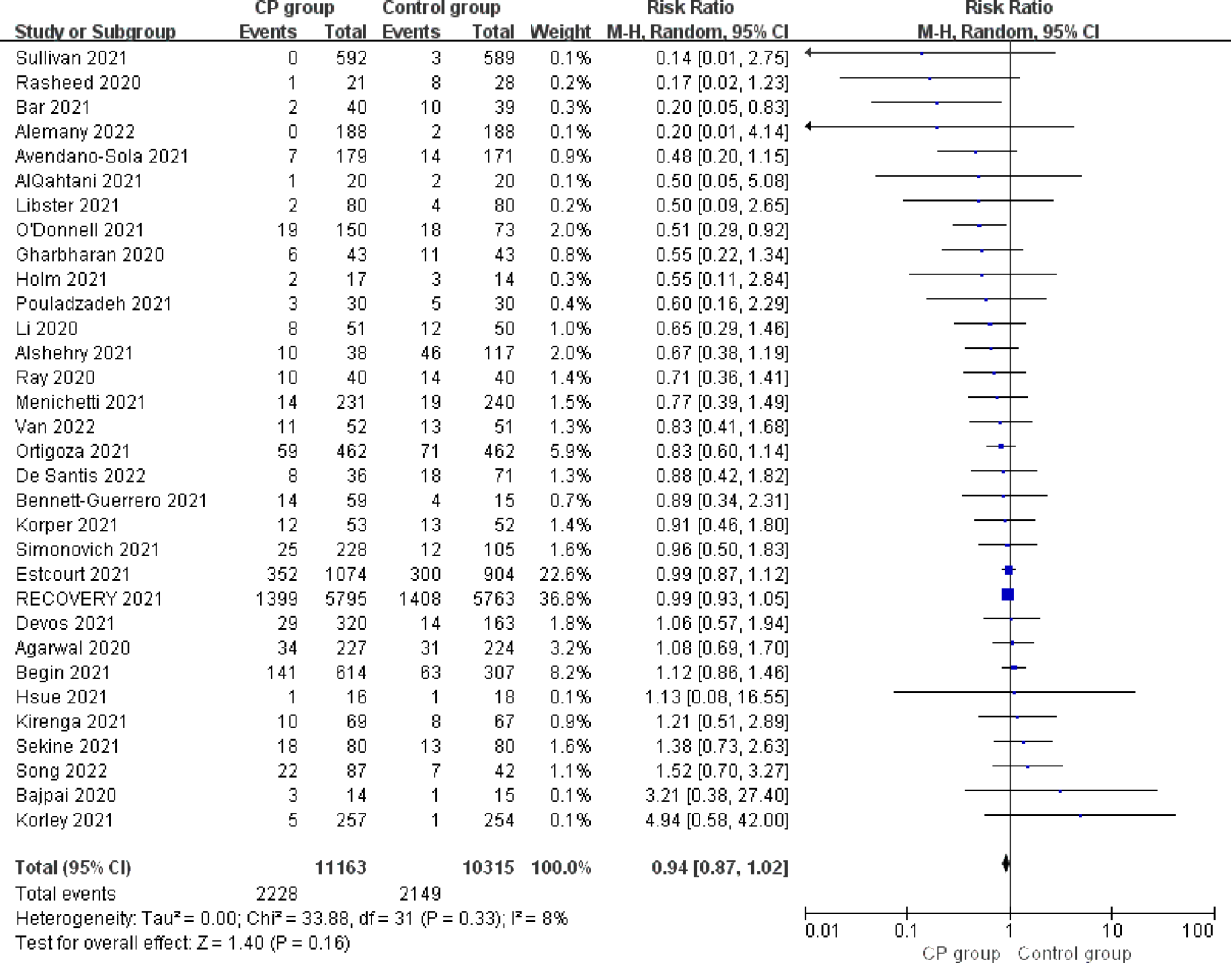
Forrest plot of the risk ratio of 28-d mortality between CCP group and control group.

The 28-d mortality was reported in all included studies. In the overall population (**Figure 3**), the 28-d mortality was 20.0% (2228/11163) in CCP group and 20.8% (2149/10315) in control group, and the risk ratio was 0.94 (95% CI 0.87-1.02; p = 0.16; I² = 8%). After excluding preprinted studies (**Additional Figure 1**), the 28-d mortality was 21.1% (2205/10474) in CCP group and 22.0% (2121/9628) in control group, without significant statistic differences (risk ratio 0.94; 95% CI 0.86-1.03; p = 0.18; I²= 12%).

Subgroup analysis suggested potential differences between double-blinded RCTs and open-label RCTs (**Additional Figure 2**). Compared to the control group, double-blinded RCTs showed reduced 28-d mortality in COVID-19 patients treated with CCP (6.5%, 136/2084 vs. 7.0%, 129/1835; risk ratio 0.78; 95% CI 0.62-0.99; p = 0.04; I² = 0%), while this association was not found in open-label RCTs (23.0%, 2092/9079 vs. 23.8%, 2020/8480; risk ratio 0.97; 95% CI 0.90-1.05; p = 0.48; I² = 7%).

Convalescent plasma neither reduced the risk for 28-d mortality in outpatients (0.6%, 7/1117 vs. 0.9%, 10/1111; risk ratio 0.63; 95% CI 0.14-2.95; p = 0.56; I² = 42%), nor in inpatients (22.1%, 2217/10046 vs 23.3%, 2140/9204; risk ratio 0.94; 95% CI 0.86-1.02; p = 0.13; I² =11%). There were no significant differences between two subgroups (**Additional Figure 3**).

Compared to the control group, CCP therapy was not associated with significantly reduced 28-d mortality in patients requiring mechanical ventilation at enrollment (35.2%, 635/1802 vs 35.7%, 613/1717; risk ratio 0.95; 95% CI 0.81-1.10; p = 0.48; I² = 40%). There was also no significant association between receiving CCP and lower 28-day mortality in patients who required non-invasive respiratory support at enrollment (20.9%, 1419/6779 vs 22.0%, 1372/6234; risk ratio 0.97; 95% CI 0.91-1.03; p = 0.34; I² = 0%) or those who did not require supplementary oxygenation at enrollment (3.9%, 58/1484 vs 5.1%, 76/1491; risk ratio 0.81; 95% CI 0.59-1.11; p = 0.19; I² = 0%).(**Additional Figure 4**)

For antibody-seronegative patients, the 28-d mortality was 32.7% (791/2419) in CCP group and 34.1% (656/1926) in control group. While for antibody-seropositive patients, the 28-d mortality was 20.2% (794/3932) in CCP group and 19.2% (673/3510) in control group. Neither patients with detectable antibodies (risk ratio 1.00; 95% CI 0.85-1.18; p = 0.96; I²=40%) nor those without detectable antibodies (risk ratio 0.94; 95% CI 0.86-1.02; p = 0.14; I²=0%) at enrollment showed reduced 28-d mortality after receiving CCP. (**Additional Figure 5**)

For patients receiving high titer CCP, the 28-d mortality was 19.9% (1682/8461) in CCP group and 20.3% (1576/7779) in control group. Receiving high titer CCP was not related to lower 28-d mortality (risk ratio 0.99; 95% CI 0.94-1.06; p = 0.83; I² = 0%). However, there was significantly reduced 28-d mortality for patients receiving low titer CCP (9.5%, 63/665 vs 13.1%, 77/587; risk ratio 0.68; 95% CI 0.55-0.92; p = 0.01; I² = 0%) compared to the control group (**Additional Figure 6**).

In the patients whose median time from symptoms onset to enrollment was no more than 7days, there were no significant differences in 28-d mortality in CCP group compared to control group (25.7%, 656/2553 vs 27.9%, 710/2549; risk ratio 0.92; 95% CI 0.84-1.01; p = 0.09; I² = 0%). For patients with more than 7 days from symptoms onset, receiving CCP treatment did not show a significant reduction in 28-d mortality (21.3%, 820/3846 vs 20.9%, 781/3745; risk ratio 0.87; 95% CI 0.59-1.26; p = 0.45; I² = 52%). There were no significant differences between the two subgroups (p=0.45; **Additional Figure 7**).

#### 3.3.2 Secondary outcomes

The length of hospital stay was reported in 11 studies, with no significant differences between the CCP group and control group (MD 0.83; 95% CI -0.24-1.90; p = 0.13; I² = 59%) (**Additional Figure 8A**). The ventilation-free days were assessed in 11 studies. Overall, the ventilation-free days were similar between the CCP group and control group (MD -0.04; 95% CI -0.74-0.67; p = 0.92; I² = 35%). (**Additional Figure 8B**)

The 14-d mortality was assessed in 6 studies. Receiving CCP was not related to significantly reduced 14-d mortality compared to the control group (5.7%, 63/1098 vs 7.0%, 65/934; risk ratio 0.88; 95% CI 0.63-1.23; p = 0.45; I² = 0%) (**Additional Figure 9**)

The deterioration and improvements of the diseases were respectively assessed in 8 studies and 9 studies. Overall, there were no significant differences in the improvement of symptoms (68.6%, 589/858 vs 65.7%, 353/537; risk ratio 1.00; 95% CI 0.94-1.07; p = 0.99; I² = 0%) and progression of diseases (27.6%, 2101/7603 vs 27.7%, 2059/7436; risk ratio 0.96; 95% CI 0.85-1.08; p = 0.49; I² = 46%) between the CCP group and control group. (**Additional Figures 10A, B**)

Initiation of mechanical ventilation was required in 20.4% (1159 of 5690) of patients receiving convalescent plasma and 21.2% (1107 of 5220) of patients with standard of care (RR 0.94, 95% CI 0.82-1.08, p = 0.38). No significant differences between CCP group and control group were observed. (**Additional Figure 11**)

#### 3.3.3 Adverse events

**Figure 4:**
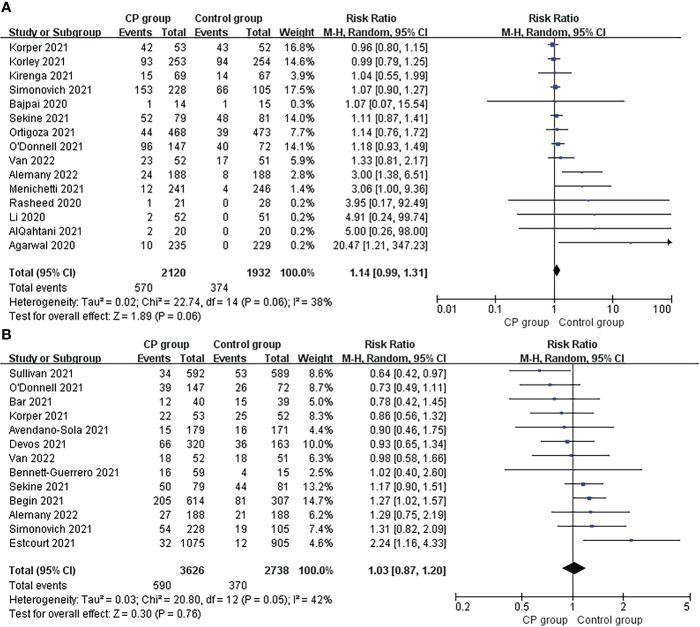
Forrest plot of the risk ratio of (A) adverse events and (B) severe adverse between CCP group and control group.

Adverse events and serious adverse events were reported in 15 studies and 13 studies, respectively. Overall, the incidence of adverse events (26.9%, 570/2120 vs 19.4%, 374/1932; risk ratio 1.14; 95% CI 0.99-1.31; p = 0.06; I² = 38%) and serious adverse events (16.3%, 590/3626 vs 13.5%, 370/2738; risk ratio 1.03; 95% CI 0.87-1.20; p = 0.76; I² = 42%) tended to be higher in the CCP group compared to the control group, though the differences did not reach statistical significance (**Figure 4A**, **B**).

### 3.4 Quality of evidence

**Figure 5:**
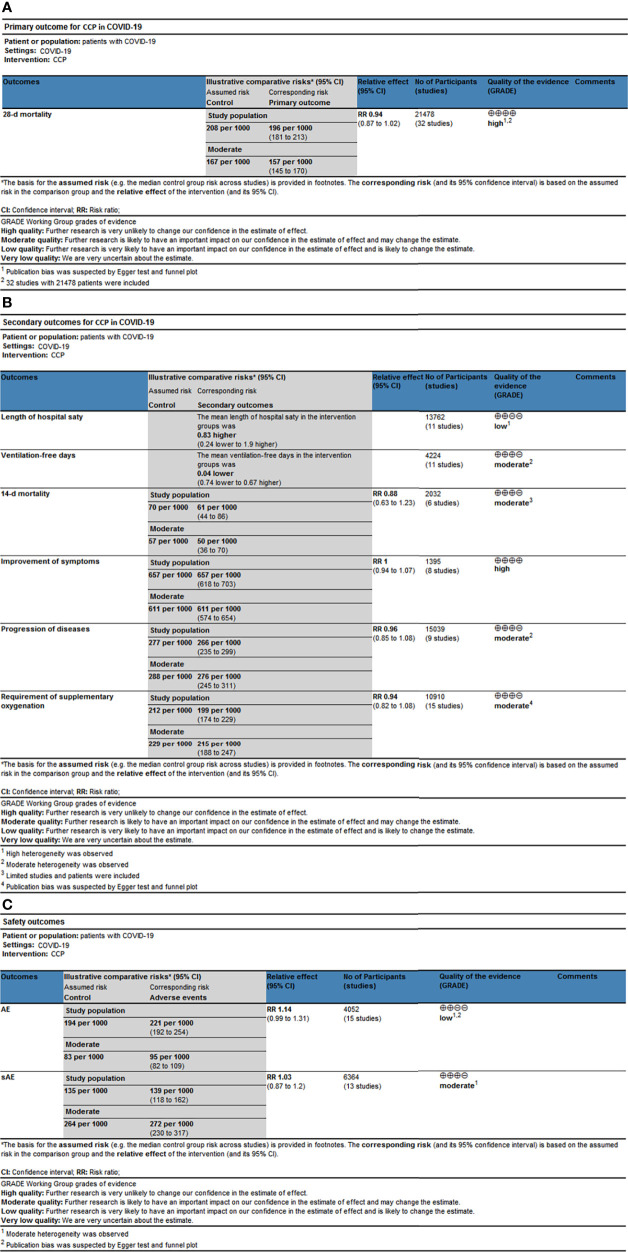
The simplified Summary of Finding of outcomes. The simplified Summary of Finding for **(A)** Primary outcomes, **(B)** Secondary outcomes and **(C)** Safety outcomes. CCP, COVID-19 convalescent plasma; 95% CI, 95% confidence interval; RR, risk ratio.

According to the GRADE assessment (**Figure 5** and **Additional File 1 Table 3**), the evidence for the effect of CCP on 28-d mortality in all patients was high, which was mainly due to the large sample size and low level of heterogeneity despite publication bias. Similarly, the evidence for the effect of CCP on 28-d mortality in inpatients was high, while it downgraded to very low for outpatients for limited patients and moderate heterogeneity. The evidence for the effect of CCP on patients receiving non-MV ventilation and high titer CCP was both moderate for publication bias (**Additional Figure 12C**). For other subgroup analysis on 28-mortaity, the evidence for the effect of CCP ranged from low to very low. For secondary outcomes, the evidence for the effect of CCP on the improvements of symptoms was high, while the evidence for the effect of CCP on the ventilation-free days, 14-d mortality, progression and requirement of supplementary oxygenation was moderate due to the moderate heterogeneity, small size of included patients or publication bias (**Additional Figures 12E, F**). The evidence for the effect of CCP on length of hospital stay was low because of the serious heterogeneity of results. The evidence for the incidence of AE and SAE was low and moderate respectively due to moderate heterogeneity and publication bias (**Additional Figures 12G, H**).

## 4 Discussion

In this meta-analysis which included 32 RCTs and 21478 patients, we found that CCP therapy was not associated with significantly reduced 28-d mortality in COVID-19 patients. Besides, receiving CCP was not related to improvements on other survival outcomes, including length of hospital stay, time without respiratory support, risk of symptoms progression and requirement of MV. In terms of safety, treatment with CCP presented a trend of higher incidence of adverse events, although the differences didn’t reach statistical significance.

At present, several therapies have been recommended by WHO (53) and IDSA (54) to treat COVID-19. For mild patients, monoclonal antibodies such as Sotrovimab (55) could reduce the risk of hospitalization, while REGEN-CoV-2 (56) might reduce mortality in patients without detectable baseline antibodies. Antivirals such as remdesivir (57, 58), favipiravir (59), molnupiravir (60) and nematvir/ritonavir (61) could reduce the risk of ventilation as well as mortality in patients at high risk of hospitalization and release the symptoms. However, in low-income countries and regions, monoclonal antibodies and antivirals might not be readily available. IDSA recommended high titer and fully qualified CCP as an alternative to monoclonal antibodies and antivirals, which was opposite to WHO guidelines that strongly recommended against CCP in mild patients due to limited clinical benefits. For critically ill patients, treatments aiming to control unbalanced inflammation were preferred to reduce the risk of ventilation and mortality, including glucocorticoids (62, 63), IL-6 receptor inhibitors (64, 65), and Baricitinib (66). In addition, glucocorticoids could also improve ventilator-free days, while IL-6 and Baritinib might play a role in reducing length of hospital stay. CCP was only recommended only in the context of clinical trials for severe COVID-19 patients, due to limited suppressive effect of CCP on inflammation and no significantly improved clinical outcomes.

Previously, there were studies suggesting the association between receiving CCP and lower 28-day mortality or less progression of diseases (67–70), while recent prospective studies and RCTs indicated that CCP could not lead to elevated antibody titer (71) or survival benefits in COVID-19 patients (72, 73). Our findings supported that COVID-19 patients might not benefit from the transfusion of CCP, which was consistent with the latest WHO and IDSA guideline (53, 54). These Inconsistencies of outcomes among these studies might be due to the heterogeneous baseline conditions of included patients (74) and the variations of interventions (75) between the CCP group and control group, especially in retrospective and observational studies. Severe COVID-19 patients were more likely to receive high titer and dosage of CCP beyond more frequent use of antiviral agents or corticosteroid, which might overestimate the efficiency of CCP.

Our study found that the administration of CCP was not related to significant improvements in 28-d mortality, length of hospital stays, ventilation-free days, or the progression of diseases. These could be due to several reasons: Firstly, most eligible studies were conducted between 2021 and 2022, when SARS-CoV-2 variants had spread widely around the world, like Delta and Omicron. Previous studies found that mutations in spike proteins, including E484A and N501Y, made these variants more likely to escape from immune recognition (76, 77), reducing the efficiency of CCP (78). Additionally, according to the analysis focused on variables associated with CCP efficacy, CCP collected from certain locations and pandemic waves couldn’t effectively neutralize the virus at other locations and waves (79). The chronological and epidemiological distance between plasma donors and receptors might lead to the mismatch in antibodies and circulating variants, resulting in further aggravation of the variants’ resistance to antibodies. Therefore, considering the attempt to standardize the plasma centrally, the efficiency of CCP might be underestimated among studies that were carried out nationally and across multiple pandemic waves.

Secondly, the majority of eligible patients in our study were no less than 7 days from symptoms onset and suffering hypoxemia at enrollment, requiring at least one type of supplementary oxygenation. Results from subgroup analysis suggested that these patients could not benefit from the CCP therapy. Indeed, for patients at the end stage of COVID-19, the pathology of lung parenchyma was mainly characterized by inflammatory infiltration and fibrosis resulting from the unbalanced pro-inflammatory response and cytokine storm, while replication of SARS-COV-2 contributed less to the damage (79, 80). The initial course of COVID-19 might be viewed as an optimal therapeutic window period for exogenous antibodies to maximize their neutralization effect (32, 81). However, our study found that there was no significantly lower 28-d mortality either in patients within 7 days from symptoms onset or those with more than 7 days. On the one hand, this could be due to the limited number of included patients in the early stages of COVID-19; on the other hand, 7 days might not be early enough to identify for potential benefit. In a multicenter retrospective study (74), administration of CCP within 3 days since symptoms onset, but not within 4 to 7 days, was related to a significantly reduced mortality. Therefore, what mattered to improve the efficiency of CCP at present was determining the appropriate therapeutic window period to identify the possible patients who might benefit from CCP therapy.

Thirdly, the variations in the standard of care among included studies might also be an important factor, especially the percentage of patients receiving corticosteroid or remdesivir which had been confirmed to be beneficial for survival. In the REMAP-CAP trial (52), up to 90% of patients in the study were treated with glucocorticoids, whereas in RECOVERY trial (35), less than 1% of patients received glucocorticoids. Similarly, the percentage of patients treated with remdesivir was more than 80% and less than 5% in the study of Bajpai et al. (28) and Agarwal et al. (25), respectively. In addition, we found that among the RCTs with placebo, receiving CCP was related to a lower risk of 28-d mortality, while this association was not observed among the open-label RCTs. Considering the weakened control of performance bias, the lack of placebo might lead to the underestimation of CCP. More double-blinded RCTs were required for further assessment.

In addition to the reasons mentioned above, we noticed that receiving CCP was related to trend of elevated incidence of adverse events compared to the control group, although the difference was not statistically significant. This might be another essential factor that should be considered when applying CCP to COVID-19 patients. However, since the funnel plot and Egger test suggested potential publication bias, the evidentiary quality of this result was low. More studies were needed for the further assessment of the safety of CCP.

Notably, we found that CCP therapy did not significantly reduce 28-day mortality regardless of whether neutralizing antibodies were detectable at enrollment. Previous studies (82) found that hypogammaglobulinemia, regardless of causes, was associated with poor survival, and the immunoglobulin replacement therapy like CCP might be beneficial for elevating level of antibodies and alleviating viremia, thus reducing symptom duration, hospital stay, and mortality. However, this relationship was not shown in our study, which might be due to the limited number of included studies. Meanwhile, the antibody seronegativity was defined as the failed detection of IgG or IgM in the included studies (29, 35, 44, 52), while the ignorance of other subtypes of antibodies like IgA might result in the misclassification of seronegative patients

Our results suggested receiving high titer CCP was not related to significantly reduced 28-d mortality. For one thing, the definition of high titer CCP remained controversial at present, which was mainly due to the inconsistent measurements across studies and the unclear cut-off value of high-and low-titer. According to the previous researches (8, 12), we defined the high titer CCP as the PRNT50 of anti-spike antibody≥1:320 or the ID50 of anti-spike antibody≥1:320, apart from the titer of anti-spike antibody≥1:640 and nAbs ≥1:40. However, this was a preliminary stratification, while the CCP titer within each subgroup might vary a lot. In the high titer group, the CCP used in Holm 2021 (36) had a median nAbs titer of 1:116 (1:40-1:1160), while the median titer of nAbs of CCP used in Sekine 2021 (49) could reach 1:320 (1:160-1:960). For another, there were no significant improvements either in the composition of antibody profile or in the avidity of antibodies after high titer CCP transfusion (nAbs 1:160-1:640), which were more likely to be related to positive clinical outcomes rather than the titer of nAbs, according to the recent study focused on the severe COVID-19 patients (83). Besides, neutralizing antibody titer showed a sharp downward trend before the death of COVID-19 patients despite the previous administration of CCP, suggesting the limited effect of high titer CCP on the composition of antibodies and preventing the failure of the immune system at the end stage of COVID-19 (83).

Notably, during the data synthesis of 28-d mortality, we noticed that the RECOVERY trial and REMAP-CAP trial accounted for 30.5% and 21.5% of the weight respectively, making our results to some extent dominated by these two studies. Previous study raised the concern that the impact of large studies might result in massive bias (81, 84), especially when the baseline conditions of patients could not be fully balanced in eligible studies. Therefore, we conducted the sensitivity analysis to assess the stability of our results, showing that the final conclusion would not be overturned even if these two RCTs were excluded simultaneously (RR 0.87; 95% CI, 0.76 to 1.00; P = 0.05; Statistical difference was set as P < 0.05). Coupled with the existence of publication bias, where both the funnel plot and Begg or Egger test had confirmed that more studies with risk ratio<1 were included, we were confident with the conclusion that CCP might not be regarded as an appropriate routine therapy for COVID-19, which was consistent with latest WHO guideline (53) and IDSA guideline (54).

There were several strengths in our study compared to previous meta-analysis (8, 12, 55, 85–88): 1. Our study was the latest meta-analysis with the data from latest RCTs; 2. More comprehensive subgroup analysis was performed, including the titer of CCP, the time from symptoms onset to enrollment and the type of control group (placebo+SOC or only SOC), which were not evaluated in previous studies; 3. We evaluated adverse events (AEs) and serious adverse events (SAEs) as the safety outcomes which were overlooked in previous studies; 4. The impact of large RCTs was weakened for larger number of eligible RCTs and patients. However, there were several limitations in our study. Firstly, although all the studies we included were RCTs, 50% of them were open-label designed, containing certain risk of bias. Subgroup analysis suggested potential differences in 28-d mortality between double-blinded RCTs and open-label RCTs, although the differences didn’t reach statistical differences. Secondly, publication bias was observed in the 28-d mortality and adverse events, which might bring certain potential bias to the results. Thirdly, the eligible RCTs involved multiple time periods and different countries or regions, suggesting that patients might be infected with multiple variants. Fourthly, 90% of the included patients required supplementary oxygenation, while only 10% of the patients were outpatients. The assessment on mild patients was insufficient. Fifthly, our study mainly focused on COVID-19 patients with normal immunity, without evaluation on patients with immunodeficiency due to lack of data and giant heterogeneous baseline conditions from normal patients. Previous studies suggested reduced risk of mortality in immune-compromised patients receiving CCP (89). Future studies were needed for further assessment. Sixthly, we didn’t assess the efficiency of CCP in low-income countries due to limited trials conducted in these countries. In fact, as a cost-effective treatment, CCP might be more suitable for these countries where antiviral and monoclonal antibodies were not readily available (54). Seventhly, the efficiency of CCP on post-exposure protection (90) was not assessed in our studies since the unconfirmed COVID-19 patients were not included according to our exclusion criteria. Eighthly, we didn’t assess the proportion of patients with negative nucleic acid test, time to the negative nucleic acid test, the proportion of patients with detectable endogenous antibodies after receiving CCP as our results. Lastly, a more comprehensive and advanced statistical modeling might be needed to better balance the baseline conditions among eligible studies, just as Troxel AB et al (12) did with a robust Bayesian framework.

## Conclusion

Compared to the control group, CCP therapy was not related to significantly improvements in 28-d mortality or other clinical outcomes in the overall COVID-19 patients. Considering the high quality of evidence, CCP should not be recognized as an appropriate routine treatment for clinicians. More double-blinded RCTs were needed to investigate the efficiency of CCP among patients in the initial stage of COVID-19, especially those who were within 3 days from symptoms onset and without detectable neutralizing antibodies at enrollment. Besides, the definition of high titer CCP required further determination.

## Data availability statement

The original contributions presented in the study are included in the article/**Supplementary Material**. Further inquiries can be directed to the corresponding author.

## Author contributions

ZQ developed the initial idea of this study and conducted a comprehensive search of databases. All authors have made their contributions for writing articles. The manuscript was drafted by ZQ, ZZ, HM, and SS. HK and ZT reviewed this article and provided suggestions for it. All of the authors have carefully examined this manuscript and agreed with the ideas presented in the article.

## Funding

This study was supported by grant 2021YFC0863600 from the Ministry of Science and Technology of the People’s Republic of China.

## Conflict of interest

The authors declare that the research was conducted in the absence of any commercial or financial relationships that could be construed as a potential conflict of interest.

## Publisher’s note

All claims expressed in this article are solely those of the authors and do not necessarily represent those of their affiliated organizations, or those of the publisher, the editors and the reviewers. Any product that may be evaluated in this article, or claim that may be made by its manufacturer, is not guaranteed or endorsed by the publisher.
