# Supplementary material for: The efficiency of convalescent plasma in COVID-19 patients: A systematic review and meta-analysis of randomized controlled clinical trials
Source: Front Immunol. 2022 Jul 28;13:964398. doi: 10.3389/fimmu.2022.964398 (PMC9366612; doi:10.3389/fimmu.2022.964398)
Supplement: Supplementary file 1 [file DataSheet_1.doc]

Additional Material 1

Table of content

**Additional Table 1** Systematic search strategy………………………………………………………………………………………………2

**Additional Table 2** Characteristics of the eligible studies………………………………………………………………………………3

**Additional Table 3** The assessment of quality of evidence…………………………………………………………………………….9

**Additional Table 1: Systematic search strategy**

| **Pubmed** | No. |
| --- | --- |
| (("COVID-19" OR "COVID-19"[MeSH Terms] OR "SARS-CoV-2" OR "sars-cov-2"[MeSH Terms] OR "Severe Acute Respiratory Syndrome Coronavirus 2" OR "coronavirus"[MeSH Terms] OR "coronavirus" OR "sars cov 2 infection"[All Fields] OR "severe acute respiratory syndrome coronavirus 2 infection"[All Fields] OR "covid 19 pandemic"[All Fields] ) AND ("convalesce"[All Fields] OR "convalesced"[All Fields] OR "convalescence"[MeSH Terms] OR "convalescence"[All Fields] OR "convalescences"[All Fields] OR "convalescent"[All Fields] OR "convalescents"[All Fields] OR "convalescing"[All Fields] OR "plasma"[MeSH Terms] OR "plasma"[All Fields] OR "plasmas"[All Fields] OR "plasma's"[All Fields] OR " passive immunization "[All Fields] OR "serotherapy"[Supplementary Concept] OR "COVID-19 serotherapy"[All Fields] OR coronavirus disease-19 serotherapy: "COVID-19 serotherapy"[Supplementary Concept] OR "COVID-19 serotherapy"[All Fields])) | 6772 |
| **Embase** |  |
| ('covid 19'/exp OR 'covid 19' OR 'covid19'/exp OR 'covid19' OR ' sars2'/exp OR ' sars2' OR ' sars-2'/exp OR ' sars-2' OR ' sars-cov-2 '/exp OR ' sars-cov-2 ' OR ' severe acute respiratory syndrome '/exp OR ' severe acute respiratory syndrome ' OR ' severe acute respiratory syndrome coronavirus 2 infection '/exp OR ' severe acute respiratory syndrome coronavirus 2 infection ' OR ' covid 19 pandemic '/exp OR 'covid 19 pandemic 9' ) AND (' convalescent '/exp OR ' convalescent ' OR ' convalescent plasma '/exp OR ' convalescent plasma ' OR ' hyperimmune plasma '/exp OR ' hyperimmune plasma ' OR ' immune plasma '/exp OR ' immune plasma ' OR ' passive immunization '/exp OR ' passive immunization ' OR ' plasma therapy '/exp OR ' plasma therapy ' OR ' serotherapy '/exp OR ' serotherapy ' OR ' passive antibody '/exp OR ' passive antibody ') | 6412 |
| **Web of Science** |  |
| (ALL=( Covid-19) OR ALL=( Covid19) OR ALL=(sars) OR ALL=(sarsa) OR ALL=(sars-2) OR ALL=(sars-cov) OR ALL=(sars-cov-2) OR ALL=(severe acute respiratory syndrome) OR ALL=(2019-ncov) OR ALL=( coronavirus)) AND (ALL=(convalescent) OR ALL=( convalescence)) AND (ALL=(convalescent plasma) OR ALL=(passive immunization) OR ALL=(plasma therapy) OR ALL=(serotherapy) OR ALL=(passive antibody transfer)) | 1875 |
| **Cochrane library** |  |
| ((Covid-19):ti,ab,kw OR (Covid19):ti,ab,kw OR (sars2):ti,ab,kw OR (sars-2):ti,ab,kw OR (sars-cov-2):ti,ab,kw OR (severe acute respiratory syndrome):ti,ab,kw OR (coronavirus):ti,ab,kw OR (severe acute respiratory syndrome coronavirus 2 infection):ti,ab,kw OR (covid 19 pandemic):ti,ab,kw) AND ((convalescent):ti,ab,kw OR (convalescent plasma):ti,ab,kw OR (hyperimmune plasma):ti,ab,kw OR (immune plasma):ti,ab,kw OR (passive immunization):ti,ab,kw OR (plasma therapy):ti,ab,kw OR (serotherapy):ti,ab,kw OR (passive antibody):ti,ab,kw) | 619 |
| **MedRxiv** |  |
| (Covid-19 OR Covid19 OR sars2 OR sars-2 OR sars-cov-2) AND (convalescent OR convalescent plasma) | 1635 |

**Additional Table 2: Characteristics of the eligible studies.**

| **Study** | **Registration ID** | **Country** | **Design** | **Inclusion criteria** | **Intervention** | **Control** | **Primary Outcome** | **WHO 7-point clinical scale at enrollment** | **Sample**  **size** | **Time from Symptom**  **onset to enrolment, days (IQR)** | **Detectable antibody** |
| --- | --- | --- | --- | --- | --- | --- | --- | --- | --- | --- | --- |
| **Agarwal**  **2020** | **CTRI/2020/04/024775** | **India** | **Open-label phase II multicentre RCT (PLACID Trial)** | **①≥18y ②RT-PCR confirmed ③Hypoxia: a. OI 200-300；b. RR≥24/min with PaO2＜93% on rest** | **400ml CCP**  **+SOC** | **SOC** | **Composite of progression to severe disease** | **WHO4 362/464**  **WHO5 102/464** | **235 CCP**  **229 SOC** | **8 (6-11)** | **348 (75%）** |
| **Alemany**  **2022** | **NCT04621123** | **Spain** | **multicentre, double-blind RCT** | **①≥50y ②RT-PCR confirmed≤5d ③Outpatient** | **250-300 ml CCP+SOC** | **250ml NS+SOC** | **1.Hospitalization rate**  **2.SARS-CoV-2 viral load** | **Not**  **reported** | **188 CCP**  **188 NS** | **4.4±1.4** | **43 (11.4%)** |
| **AlQahtani 2021** | **NCT04356534** | **Bahrain** | **Open-label RCT** | **①≥21y ②PCR confirmed ③Hypoxia: a.SaO_2_＜92% on room air; b.PO_2_ <60 mmHg; c. OI＜300 requiring oxygen therapy"** | **400ml CCP**  **+SOC** | **SOC** | **Requirement for invasive ventilation** | **WHO4 36/40**  **WHO5 4/40** | **20 CCP**  **20 SOC** | **Not**  **reported** | **Not reported** |
| **AlShehry**  **2021** | **NCT04347681** | **Saudi Arabia** | **Open-label, two arms, phase II national trial** | **①≥18y ②RT-PCR confirmed ③Hypoxia: a. Dyspnea b. RR≥30 c. PaO2＜93% d. OI＜300 e. Requiring ICU** | **300ml CCP**  **+SOC** | **SOC** | **1. ICU length of stay**  **2. Safety of CP& Serious adverse reactions.** | **WHO5 60/164**  **WHO6 104/164** | **40 CCP**  **124 SOC** | **Not**  **reported** | **Not reported** |
| **Avendano-**  **Sola 2021** | **NCT04345523** | **Spain** | **multicenter open-label RCT**  **（ConPlas-19）** | **①≥18y ②RT-PCR confirmed ③Hypoxia: a.PaO2＜94% on room air，requiring supplemental oxygen** | **250-300 ml CCP+SOC** | **SOC** | **Category Changes in the "WHO 7-Ordinal Scale"** | **WHO3 74/250**  **WHO4 176/250** | **179 CCP**  **171 SOC** | **6(4–7)** | **109 (31.1%)** |
| **Bajpai**  **2020** | **NCT04346446** | **India** | **open-label, phase II RCT** | **①18-65y ②RT-PCR confirmed ③hypoxia：a.SaO2＜93% in resting state b. OI ＜300 but ≥150 c. RR＞30/min** | **500ml CCP**  **+SOC** | **SOC** | **Proportion of patients remaining free of MV** | **Not**  **reported** | **14 CCP**  **15 SOC** | **Not**  **reported** | **Not reported** |
| **Bar 2021** | **NCT04397757** | **United States** | **open-label RCT** | **①≥18y ②RT-PCR confirmed ③Hypoxia: a.PaO2＜93% on room air b. Requiring supplemental oxygen c.RR≥30/min"** | **400ml CCP**  **+SOC** | **SOC** | **1. Participants With Serious Adverse Events.**  **2.Clinical Severity Score** | **WHO3 4/80**  **WHO4 43/80**  **WHO5 33/80** | **40 CCP**  **40 SOC** | **6 (4–9)** | **33 (41.3%)** |
| **Begin 2021** | **NCT04348656** | **Brazil, Canada, United States** | **multicenter, open-label RCT（CONCOR-1 trail）** | **①≥18y ②RT-PCR confirmed ③requiring supplemental oxygen** | **500ml CCP**  **+SOC** | **SOC** | **Number of Participants Who were intubated or Died** | **Not reported** | **548 CCP**  **303 SOC** | **8 (5, 10)** | **Not reported** |
| **Bennett-**  **Guerrero 2021** | **NCT04344535** | **United States** | **double-blind RCT** | **①≥18y**  **②RT-PCR confirmed** | **450-550ml CCP+SOC** | **450-550ml SP+SOC** | **28-Day Ventilator Free Days** | **WHO3 21/74**  **WHO4 34/74**  **WHO5 5/74**  **WHO6 14/74** | **59 CCP**  **15 SP** | **9 (6–18)** | **56 (75.7%)** |
| **De Santis 2022** | **RBR-7f4mt9f** | **Brazil** | **multicenter open-label RCT** | **①18-80y ②RT-PCR confirmed ③within 10 days of initial symptoms ④Hypoxia: a. OI≤300 b.PaO2＜93% on room air c. MV required** | **1800ml CCP+SOC** | **SOC** | **Survival rate in each group on day 30 of intubation or diagnosis of respiratory failure.** | **WHO6 107/107** | **36 CCP**  **71 SOC** | **8 (7-10)** | **87 (81.5%)** |
| **Devos 2021** | **NCT04429854** | **Belgium** | **open-label, multicentre RCT** | **①≥18y ②RT-PCR confirmed ③hospitalized ④ not receiving MV** | **400-500ml CCP+SOC** | **SOC** | **Patients requiring MV or death** | **WHO3 57/483**  **WHO≥4 425/483** | **320 CCP**  **163 SOC** | **6 (3–8)** | **Not reported** |
| **Estcourt**  **2021** | **NCT02735707** | **Australia, Belgium, Canada, et al** | **international, multicenter, open-label RCT** | **①≥18y ②RT-PCR confirmed ③severely or critically ill: a.ICU admission b. Need of MV，HFNC and FiO2≥40% or cardiovascular support** | **550±150 mL CCP +SOC** | **SOC** | **1.All-cause mortality**  **2.Days alive and not receiving organ support in ICU** | **WHO4 3/1987**  **WHO5 1336/1987**  **WHO6 648/1987** | **1078 CCP**  **909 SOC** | **Not reported** | **1012 (50.9%)** |
| **Gharbharan 2020** | **NCT04342182** | **Netherlands** | **nationwide multicenter open-label RCT** | **①＞18y**  **②RT-PCR confirmed** | **300-600ml CCP+SOC** | **SOC** | **Overall mortality until discharge or 60 days after admission** | **WHO4 8/86**  **WHO5 65/86**  **WHO6 13/86** | **43 CCP**  **43 SOC** | **11 (6–16)** | **69 (80%)** |
| **Holm**  **2021** | **NCT04600440** | **Sweden** | **open-label RCT** | **①≥18y ②RT-PCR and CT confirmed ③supplemental oxygen"** | **600-750ml CCP+SOC** | **SOC** | **Number of days in need of oxygen** | **WHO3 2/31**  **WHO4 26/31**  **WHO6 3/31** | **17 CCP**  **14 SOC** | **Not reported** | **Not reported** |
| **Hsue**  **2021** | **NCT04421404** | **United States** | **double-blind RCT** | **①≥18y ②RT-PCR and chest imaging ③PaO2＜95% on room air"** | **250ml CCP+SOC** | **250ml SP+SOC** | **MV or Death Endpoint** | **Not reported** | **16 CCP**  **18 SP** | **Not reported** | **Not reported** |
| **Kirenga 2021** | **NCT04542941** | **Uganda** | **open-label RCT** | **①RT-PCR confirmed ②irrespective of disease severity"** | **500ml CCP+SOC** | **SOC** | **Time to viral clearance (RT-PCR negativity)** | **Not reported** | **69 CCP**  **67 SOC** | **7 (5–10)** | **Not reported** |
| **Korley**  **2021** | **NCT04355767** | **United States** | **phase 3, multicenter double-blind RCT** | **①≥18y ②confirmed by nucleic acid assay**  **③Within 7 days of symptoms ④outpatients"** | **250ml CCP+SOC** | **250ml NS+SOC** | **Number of Patients With Disease Progression** | **WHO3 263/511**  **WHO4 248/511** | **257 CCP**  **254 NS** | **4 (2–5)** | **Not reported** |
| **Korper**  **2021** | **NCT04433910** | **Germany** | **multicenter, open-label RCT** | **①18-75y ②RT-PCR confirmed ③Hypoxia: a. need of respiratory support b. RR≥30/min c. admission of ICU** | **750-1000ml CCP+SOC** | **SOC** | **Composite endpoint of survival and no longer fulfilling criteria of severe COVID-19.** | **Not reported** | **53 CCP**  **52 SOC** | **7 (5–10.5)** | **75 (71.8%)** |
| **Li 2020** | **ChiCTR2000029757** | **China** | **Open-label, multicenter RCT** | **①≥18y ②RT-PCR and chest imaging confirmed ③Hypoxia: a. OI 100-300 b. RR≥30/min c.PaO2＜93% on resting d.MV or organ failure or ICU"** | **4-13ml/Kg CCP+SOC** | **SOC** | **the number of days between randomized grouping and clinical improvement within 28 days admission** | **WHO3 7/103**  **WHO4 14/103**  **WHO5 46/103**  **WHO6 36/103** | **52 CCP**  **51 SOC** | **30 (19-38)** | **Not reported** |
| **Libster**  **2021** | **NCT04479163** | **Argentina** | **Double-blind RCT** | **①≥65y ②RT-PCR confirmed③ at least one of each signs or symptoms of COVID-19** | **250ml CCP+SOC** | **250ml NS+SOC** | **Development of severe respiratory disease** | **WHO3 4/160**  **WHO4 46/160**  **WHO5 70/160**  **WHO6 40/160** | **80 CCP**  **80 SOC** | **1.6±0.6** | **Not reported** |
| **Menichetti 2021** | **NCT04716556** | **Italy** | **multicenter, national, open-label RCT** | **①≥18y ②RT-PCR and radiologically confirmed**  **③Hypoxia: OI 200-300** | **200-600ml CCP+SOC** | **SOC** | **Number of patients who meet invasive MV or death** | **Not reported** | **232 CP 241 SOC** | **7 (5-9)** | **112 (23.8%)** |
| **O'Donnell 2021** | **NCT04359810** | **Brazil, United States** | **investigator-**  **initiated, double-blind RCT** | **①≥18y ②RT-PCR and CT confirmed ③Hypoxia: a.PaO2＜93% on room air b. need of supplemental oxygen, MV or ECMO** | **200-250ml CCP+SOC** | **200-250ml SP+SOC** | **Day 28 severity** | **WHO4 145/219**  **WHO5 51/219**  **WHO6 23/219** | **147 CCP**  **72 SP** | **10 (7–13)** | **Not reported** |
| **Ortigoza 2021** | **NCT04364737** | **United States** | **investigator-initiated, double-blind RCT** | **①≥18y ②RT-PCR confirmed ③requirement of noninvasive oxygen supplementation "** | **250ml CCP+SOC** | **250ml NS+SOC** | **Score on the WHO 11-point Ordinal Scale at 14 Days** | **WHO3 47/941**  **WHO4-5 771/941**  **WHO6 123/941** | **468 CCP**  **473 NS** | **7 (4-9)** | **Not reported** |
| **Pouladzadeh**  **2021** | **IRCT20200310046736N1** | **Iran** | **hospital-based, parallel-group RCT** | **①≥18y ②RT-PCR、CT and symptoms confirmed ③WHO score > 4 ④Hypoxia: PaO2＜93%"** | **500-1000 ml CCP + SOC** | **SOC** | **The improvement in the levels of cytokine storm indices** | **WHO4 17/60**  **WHO5 43/60** | **30 CCP**  **30 SOC** | **Not reported** | **Not reported** |
| **Rasheed**  **2020** | **BKH-CT-012** |  | **Open-label, multicenter RCT** | **①≥18 y ②SpO2<90% in resting state. ③at their first 3 days in RCU requiring ventilators** | **400ml CCP+SOC** | **SOC** | **All cause mortality at 30 days** | **Not reported** | **21 CCP**  **28 SOC** | **Not reported** | **Not reported** |
| **Ray 2020** | **CTRI/2020/05/025209** | **India** | **single center open-label phase II RCT** | **①≥18y ②RT-PCR confirmed ③Severely ill: a. RR≥30 b.SpO2＜90% on room air c. ARDS：OI200-300 or OI 100-200 not on MV** | **400ml CCP+SOC** | **SOC** | **All cause mortality at 30 days** | **WHO4 60/80**  **WHO5 20/80** | **40 CCP**  **40 SOC** | **Not reported** | **Not reported** |
| **RECOVERY**  **2021** | **NCT04381936** | **Ghana,**  **India, Indonesia, et al** | **investigator-**  **initiated, open-label**  **RCT** | **①any age ②suspected or laboratory-confirmed SARS-CoV-2** | **400-700ml CCP+SOC** | **SOC** | **All-cause mortality** | **Not reported** | **5795 CCP**  **5763 SOC** | **9 (6–12)** | **5895（51.0%）** |
| **Sekine**  **2021** | **NCT04547660** | **Brazil** | **investigator-initiated, parallel arm, open-label, RCT** | **①≥18y ②RT-PCR confirmed ③Hypoxia: a. RR≥30 b.SpO2≤93% on room air c. Supplemental oxygen** | **600ml CCP+SOC** | **SOC** | **Clinical improvement** | **WHO4 40/154**  **WHO5 50/154**  **WHO6 64/154** | **75 CCP**  **79 SOC** | **Not reported** | **128（83.1%）** |
| **Simonovich**  **2021** | **NCT04383535** | **Argentina** | **double-blind, multicenter RCT** | **①≥18y ②RT-PCR confirmed ③Hypoxia: a.PaO2＜93% on room air b. OI＜300 c. SOFA/mSOFA ≥2 above baseline** | **500ml CCP+SOC** | **500ml NS+SOC** | **Clinical status during follow-up at 30th day** | **WHO4 216/333**  **WHO5 83/333**  **WHO6 34/333** | **228 CCP**  **105 NS** | **8 (5–10)** | **180（54.0%）** |
| **Song 2022** | **NCT04415086** | **Brazil** | **multicenter, open-label RCT** | **①≥18y ②RT-PCR and tomography confirmed ③Hypoxia: a.>3L in catheter/mask b.FiO2 >25% in the Venturi mask c.OI＜300 d. intubation ≤48h** | **200mL or 400mL CCP+SOC** | **SOC** | **Time elapsed until clinical improvement or hospital discharge** | **WHO4 52/129**  **WHO5 23/129**  **WHO6 54/129** | **87 CCP**  **42 SOC** | **8 (6-9)** | **92（71%）** |
| **Sullivan 2021** | **NCT04373460** | **United States** | **multicenter, double-blind RCT** | **①≥18y ②RT-PCR confirmed ③outpatients** | **250ml CCP+SOC** | **250ml SP+SOC** | **1.Incidence of hospitalization or death**  **2. Incidence of treatment-related adverse events** | **Not reported** | **592 CCP**  **589 SP** | **5 (4-7)** | **Not reported** |
| **Van 2022** | **NCT04373460** | **South Africa** | **phase III**  **double-blind RCT** | **①≥18y ②RT-PCR confirmed ③Hypoxia: a.PaO2＜94% on room air b. non-invasive oxygen** | **200-250ml CCP+SOC** | **200-250 NS+SOC** | **Clinical Improvement** | **Not reported** | **52 CCP**  **51 SOC** | **8 (6–10)** | **Not reported** |

**CCP, COVID-19 convalescent plasma; SOC, standard of care, may included hydroxychloroquine, remdesivir, lopinavir/ritonavir, oseltamivir，broad spectrum antibiotics or immunomodulators, and all other possible supportive treatments; NS, normal saline; SP standard plasma; RR, respiratory rate; OI, oxygenation index, equals to FiO2/PaO2; MV, mechanical ventilation; HFNC, High Flow Nasal Cannula.**

**Additional Table 3: The assessment of quality of evidence.**

| **Primary outcome** | | | | | |  |
| --- | --- | --- | --- | --- | --- | --- |
| **Patient or population:** patients with COVID-19 | | | | | |  |
| **Outcomes** | **Illustrative comparative risks* (95% CI)** | | **Relative effect (95% CI)** | **No of Participants (studies)** | **Quality of the evidence (GRADE)** |  |
|  | Assumed risk | Corresponding risk |  |  |  |  |
|  | **Control** | **Primary outcome** |  |  |  |  |
| **28-d mortality** | **Study population** | | **RR 0.94**  (0.87 to 1.02) | 21478 (32 studies) | ⊕⊕⊕⊕ **high**^1,2^ |  |
|  | **208 per 1000** | **196 per 1000** (181 to 213) |  |  |  |  |
|  | **Moderate** | |  |  |  |  |
|  | **167 per 1000** | **157 per 1000** (145 to 170) |  |  |  |  |
| **28-mortality - Outpatients** | **Study population** | | **RR 0.63**  (0.14 to 2.95) | 2228 (4 studies) | ⊕⊝⊝⊝ **very low**^3,4^ |  |
|  | **9 per 1000** | **6 per 1000** (1 to 27) |  |  |  |  |
|  | **Moderate** | |  |  |  |  |
|  | **8 per 1000** | **5 per 1000** (1 to 24) |  |  |  |  |
| **28-mortality - Inpatients** | **Study population** | | **RR 0.94**  (0.86 to 1.02) | 19250 (28 studies) | ⊕⊕⊕⊕ **high**^1,5^ |  |
|  | **233 per 1000** | **219 per 1000** (200 to 237) |  |  |  |  |
|  | **Moderate** | |  |  |  |  |
|  | **210 per 1000** | **197 per 1000** (181 to 214) |  |  |  |  |
| **28-d mortality - MV** | **Study population** | | **RR 0.95**  (0.81 to 1.1) | 3519 (8 studies) | ⊕⊕⊝⊝ **low**^1,3^ |  |
|  | **357 per 1000** | **339 per 1000** (289 to 393) |  |  |  |  |
|  | **Moderate** | |  |  |  |  |
|  | **375 per 1000** | **356 per 1000** (304 to 413) |  |  |  |  |
| **28-d mortality - non-MV** | **Study population** | | **RR 0.97**  (0.91 to 1.03) | 13013 (15 studies) | ⊕⊕⊕⊝ **moderate**^1^ |  |
|  | **220 per 1000** | **213 per 1000** (200 to 227) |  |  |  |  |
|  | **Moderate** | |  |  |  |  |
|  | **114 per 1000** | **111 per 1000** (104 to 117) |  |  |  |  |
| **28-d mortality - No supplementary oxygenation** | **Study population** | | **RR 0.81**  (0.59 to 1.11) | 2975 (5 studies) | ⊕⊕⊝⊝ **low**^1,4^ |  |
|  | **51 per 1000** | **41 per 1000** (30 to 57) |  |  |  |  |
|  | **Moderate** | |  |  |  |  |
|  | **11 per 1000** | **9 per 1000** (6 to 12) |  |  |  |  |
| **28-d mortality - Antibody-seronegative** | **Study population** | | **RR 0.94**  (0.86 to 1.02) | 4345 (4 studies) | ⊕⊕⊝⊝ **low**^4^ |  |
|  | **341 per 1000** | **320 per 1000** (293 to 347) |  |  |  |  |
|  | **Moderate** | |  |  |  |  |
|  | **256 per 1000** | **241 per 1000** (220 to 261) |  |  |  |  |
| **28-d mortality - Antibody-seropositive** | **Study population** | | **RR 1**  (0.85 to 1.18) | 7442 (4 studies) | ⊕⊝⊝⊝ **very low**^3,4^ |  |
|  | **192 per 1000** | **192 per 1000** (163 to 226) |  |  |  |  |
|  | **Moderate** | |  |  |  |  |
|  | **232 per 1000** | **232 per 1000** (197 to 274) |  |  |  |  |
| **28-d mortality - No more than 7 days from symptoms onset** | **Study population** | | **RR 0.92**  (0.84 to 1.01) | 5102 (3 studies) | ⊕⊕⊝⊝ **low**^4^ |  |
|  | **279 per 1000** | **256 per 1000** (234 to 281) |  |  |  |  |
|  | **Moderate** | |  |  |  |  |
|  | **263 per 1000** | **242 per 1000** (221 to 266) |  |  |  |  |
| **28-d mortality - More than 7 days from symptoms onset** | **Study population** | | **RR 0.87**  (0.59 to 1.26) | 7591 (3 studies) | ⊕⊝⊝⊝ **very low**^4,6^ |  |
|  | **209 per 1000** | **181 per 1000** (123 to 263) |  |  |  |  |
|  | **Moderate** | |  |  |  |  |
|  | **212 per 1000** | **184 per 1000** (125 to 267) |  |  |  |  |
| **28-d mortality - High titer CCP** | **Study population** | | **RR 0.99**  (0.93 to 1.05) | 16590 (17 studies) | ⊕⊕⊕⊝ **moderate**^1^ |  |
|  | **200 per 1000** | **198 per 1000** (186 to 210) |  |  |  |  |
|  | **Moderate** | |  |  |  |  |
|  | **163 per 1000** | **161 per 1000** (152 to 171) |  |  |  |  |
| **28-d mortality - Low titer CCP** | **Study population** | | **RR 0.71**  (0.51 to 0.99) | 902 (4 studies) | ⊕⊕⊝⊝ **low**^4^ |  |
|  | **151 per 1000** | **108 per 1000** (77 to 150) |  |  |  |  |
|  | **Moderate** | |  |  |  |  |
|  | **248 per 1000** | **176 per 1000** (126 to 246) |  |  |  |  |
| **28-d mortality - Undivided titer CCP** | **Study population** | | **RR 0.92**  (0.76 to 1.12) | 3648 (6 studies) | ⊕⊕⊝⊝ **low**^3,4^ |  |
|  | **250 per 1000** | **230 per 1000** (190 to 280) |  |  |  |  |
|  | **Moderate** | |  |  |  |  |
|  | **205 per 1000** | **189 per 1000** (156 to 230) |  |  |  |  |
| *The basis for the **assumed risk** (e.g. the median control group risk across studies) is provided in footnotes. The **corresponding risk** (and its 95% confidence interval) is based on the assumed risk in the comparison group and the **relative effect** of the intervention (and its 95% CI).  **CI:** Confidence interval; **RR:** Risk ratio; | | | | | | |
| GRADE Working Group grades of evidence **High quality:** Further research is very unlikely to change our confidence in the estimate of effect.  **Moderate quality:** Further research is likely to have an important impact on our confidence in the estimate of effect and may change the estimate. **Low quality:** Further research is very likely to have an important impact on our confidence in the estimate of effect and is likely to change the estimate. **Very low quality:** We are very uncertain about the estimate. | | | | | | |
| ^1^ Publication bias was suspected by Egger test and funnel plot ^2^ 32 studies with 21478 patients were included ^3^ Moderate heterogeneity was observed ^4^ Limited studies and patients were included ^5^ 28 studies with nearly 20000 patients were included ^6^ High heterogeneity was observed | | | | | | |

| **Secondary outcomes** | | | | | |
| --- | --- | --- | --- | --- | --- |
| **Patient or population:** patients with COVID-19 | | | | | |
| **Outcomes** | **Illustrative comparative risks* (95% CI)** | | **Relative effect (95% CI)** | **No of Participants (studies)** | **Quality of the evidence (GRADE)** |
|  | Assumed risk | Corresponding risk |  |  |  |
|  | **Control** | **Secondary outcomes** |  |  |  |
| **Length of hospital saty** |  | The mean length of hospital saty in the intervention groups was **0.83 higher** (0.24 lower to 1.9 higher) |  | 13762 (11 studies) | ⊕⊕⊝⊝ **low**^1^ |
| **Ventilation-free days** |  | The mean ventilation-free days in the intervention groups was **0.04 lower** (0.74 lower to 0.67 higher) |  | 4224 (11 studies) | ⊕⊕⊕⊝ **moderate**^2^ |
| **14-d mortality** | **Study population** | | **RR 0.88**  (0.63 to 1.23) | 2032 (6 studies) | ⊕⊕⊕⊝ **moderate**^3^ |
|  | **70 per 1000** | **61 per 1000** (44 to 86) |  |  |  |
|  | **Moderate** | |  |  |  |
|  | **57 per 1000** | **50 per 1000** (36 to 70) |  |  |  |
| **Improvement of symptoms** | **Study population** | | **RR 1**  (0.94 to 1.07) | 1395 (8 studies) | ⊕⊕⊕⊕ **high** |
|  | **657 per 1000** | **657 per 1000** (618 to 703) |  |  |  |
|  | **Moderate** | |  |  |  |
|  | **611 per 1000** | **611 per 1000** (574 to 654) |  |  |  |
| **Progression of diseases** | **Study population** | | **RR 0.96**  (0.85 to 1.08) | 15039 (9 studies) | ⊕⊕⊕⊝ **moderate**^2^ |
|  | **277 per 1000** | **266 per 1000** (235 to 299) |  |  |  |
|  | **Moderate** | |  |  |  |
|  | **288 per 1000** | **276 per 1000** (245 to 311) |  |  |  |
| **Requirement of supplementary oxygenation** | **Study population** | | **RR 0.94**  (0.82 to 1.08) | 10910 (15 studies) | ⊕⊕⊕⊝ **moderate**^4^ |
|  | **212 per 1000** | **199 per 1000** (174 to 229) |  |  |  |
|  | **Moderate** | |  |  |  |
|  | **229 per 1000** | **215 per 1000** (188 to 247) |  |  |  |
| *The basis for the **assumed risk** (e.g. the median control group risk across studies) is provided in footnotes. The **corresponding risk** (and its 95% confidence interval) is based on the assumed risk in the comparison group and the **relative effect** of the intervention (and its 95% CI).  **CI:** Confidence interval; **RR:** Risk ratio; | | | | | |
| GRADE Working Group grades of evidence **High quality:** Further research is very unlikely to change our confidence in the estimate of effect.  **Moderate quality:** Further research is likely to have an important impact on our confidence in the estimate of effect and may change the estimate. **Low quality:** Further research is very likely to have an important impact on our confidence in the estimate of effect and is likely to change the estimate. **Very low quality:** We are very uncertain about the estimate. | | | | | |
| ^1^ High heterogeneity was observed ^2^ Moderate heterogeneity was observed ^3^ Limited studies and patients were included ^4^ Publication bias was suspected by Egger test and funnel plot | | | | | |

| **Safety outcomes** | | | | | |
| --- | --- | --- | --- | --- | --- |
| **Patient or population:** patients with COVID-19 | | | | | |
| **Outcomes** | **Illustrative comparative risks* (95% CI)** | | **Relative effect (95% CI)** | **No of Participants (studies)** | **Quality of the evidence (GRADE)** |
|  | Assumed risk | Corresponding risk |  |  |  |
|  | **Control** | **Adverse events** |  |  |  |
| **AE** | **Study population** | | **RR 1.14** (0.99 to 1.31) | 4052 (15 studies) | ⊕⊕⊝⊝ **low**^1,2^ |
|  | **194 per 1000** | **221 per 1000** (192 to 254) |  |  |  |
|  | **Moderate** | |  |  |  |
|  | **83 per 1000** | **95 per 1000** (82 to 109) |  |  |  |
| **sAE** | **Study population** | | **RR 1.03** (0.87 to 1.2) | 6364 (13 studies) | ⊕⊕⊕⊝ **moderate**^1^ |
|  | **135 per 1000** | **139 per 1000** (118 to 162) |  |  |  |
|  | **Moderate** | |  |  |  |
|  | **264 per 1000** | **272 per 1000** (230 to 317) |  |  |  |
| *The basis for the **assumed risk** (e.g. the median control group risk across studies) is provided in footnotes. The **corresponding risk** (and its 95% confidence interval) is based on the assumed risk in the comparison group and the **relative effect** of the intervention (and its 95% CI).  **CI:** Confidence interval; **RR:** Risk ratio; | | | | | |
| GRADE Working Group grades of evidence **High quality:** Further research is very unlikely to change our confidence in the estimate of effect.  **Moderate quality:** Further research is likely to have an important impact on our confidence in the estimate of effect and may change the estimate. **Low quality:** Further research is very likely to have an important impact on our confidence in the estimate of effect and is likely to change the estimate. **Very low quality:** We are very uncertain about the estimate. | | | | | |
| ^1^ Moderate heterogeneity was observed ^2^ Publication bias was suspected by Egger test and funnel plot | | | | | |
