# Supplementary material for: The efficiency of convalescent plasma in COVID-19 patients: A systematic review and meta-analysis of randomized controlled clinical trials
Source: Front Immunol. 2022 Jul 28;13:964398. doi: 10.3389/fimmu.2022.964398 (PMC9366612; doi:10.3389/fimmu.2022.964398)
Supplement: Supplementary file 2 [file DataSheet_2.doc]

Additional Material 2

Table of content

**Additional Figure 1** Forrest plot of the risk ratio of 28-d mortality between CCP group and control group in the subgroups of preprinted studies and published studies…………….....................................................................**2**

**Additional Figure 2** Forrest plot of the risk ratio of 28-d mortality between CCP group and control group in the subgroups of RCTs with or without placebo……………………………………………………………………………….…………**3**

**Additional Figure 3** Forrest plot of the risk ratio of 28-d mortality between CCP group and control group in the subgroups of outpatients and inpatients…………………………………......................................................................**4**

**Additional Figure 4** Forrest plot of the risk ratio of 28-d mortality between CCP group and control group in the subgroups of patients requiring MV, non-invasive ventilation or no supplementary oxygenation at enrollment……………………………………………………………………………………………………………………………………………….…..**5**

**Additional Figure 5** Forrest plot of the risk ratio of 28-d mortality between CCP group and control group in the subgroups of antibody-seronegative and antibody-seropositive patients…………………………………………………….**.6**

**Additional Figure 6** Forrest plot of the risk ratio of 28-d mortality between CCP group and control group in the subgroups of patients receiving high titer CCP, low titer CCP or undivided titer of CCP………………………………….**7**

**Additional Figure 7** Forrest plot of the risk ratio of 28-d mortality between CCP group and control group in the subgroups of patients with ≤7 days or ＞7 day from symptoms onset to enrollment……………………………….**8**

**Additional Figure 8** Forrest plot of the mean difference of Length of hospital stay and Ventilation-free days between CCP group and control group………………………………………………………………………………………….………….…..**9**

**Additional Figure 9** Forrest plot of the risk ratio of 14-d mortality between CCP group and control group....**10**

**Additional Figure 10** Forrest plot of the risk ratio of Improvements of symptoms and progression of diseases between CCP group and control group……………………..……………………………………………………………………………….….**11**

**Additional Figure 11** Forrest plot of the risk ratio of requirement of mechanical ventilation between CCP group and control group…..…………………………………………………………………………………………………………………....…...**12**

**Additional Figure 12** Funnel plots of the effects estimates of the outcomes……………………………………………...…**13**

**
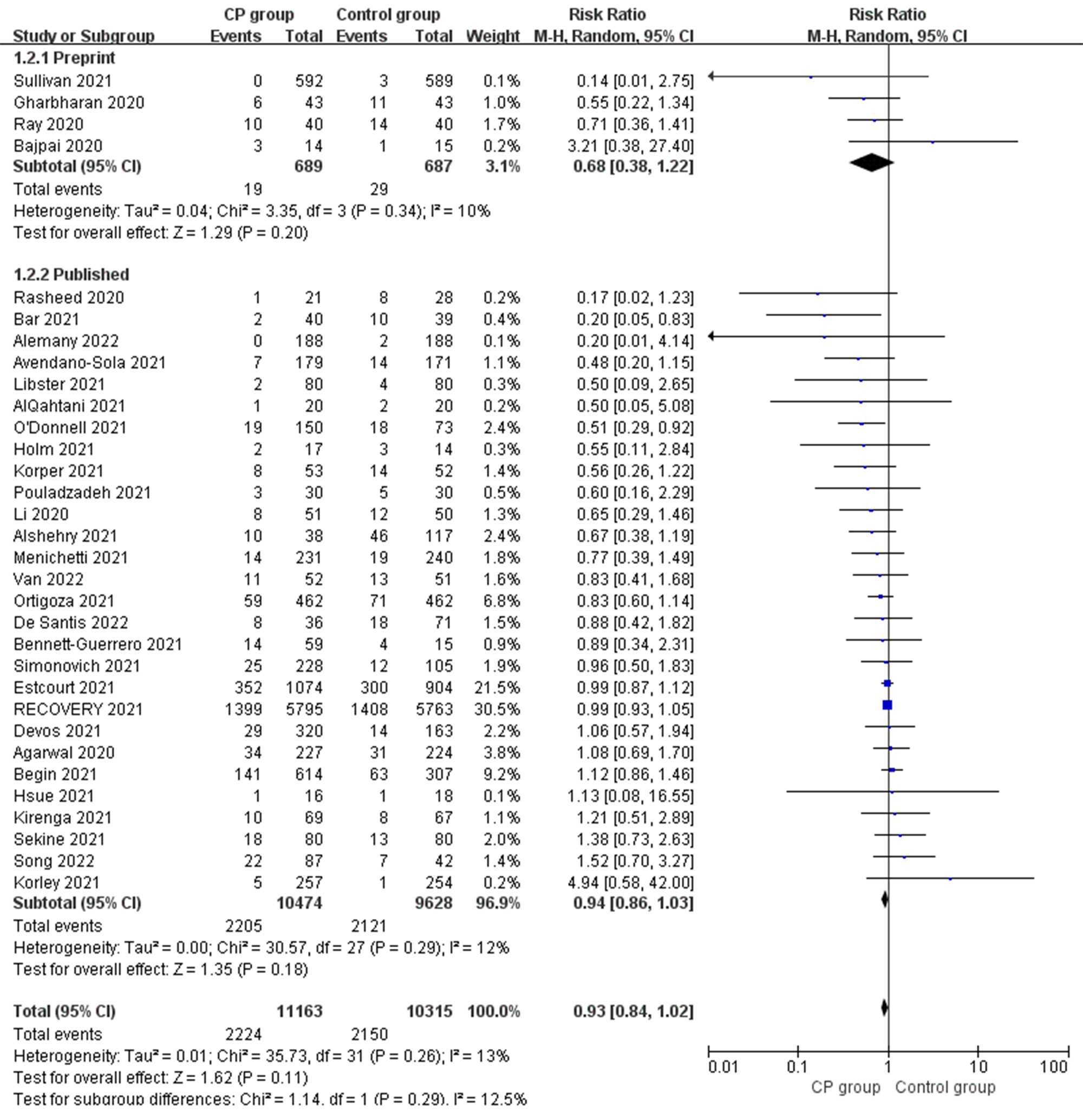
**

**Additional Figure 1:** Forrest plot of the risk ratio of 28-d mortality between CCP group and control group in the subgroups of preprinted studies and published studies.


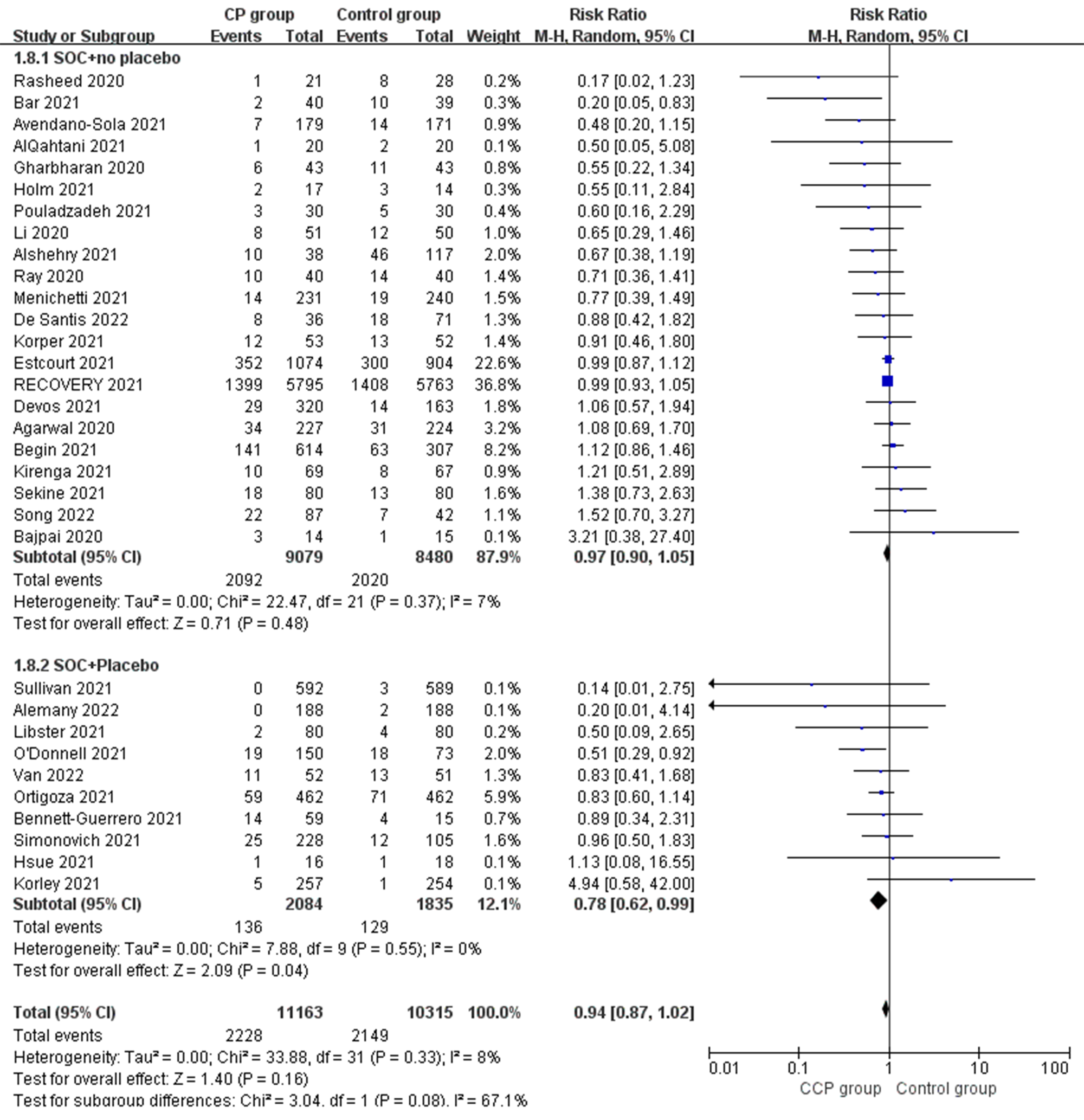


**Additional Figure 2**: Forrest plot of the risk ratio of 28-d mortality between CCP group and control group in the subgroups of RCTs with or without placebo.


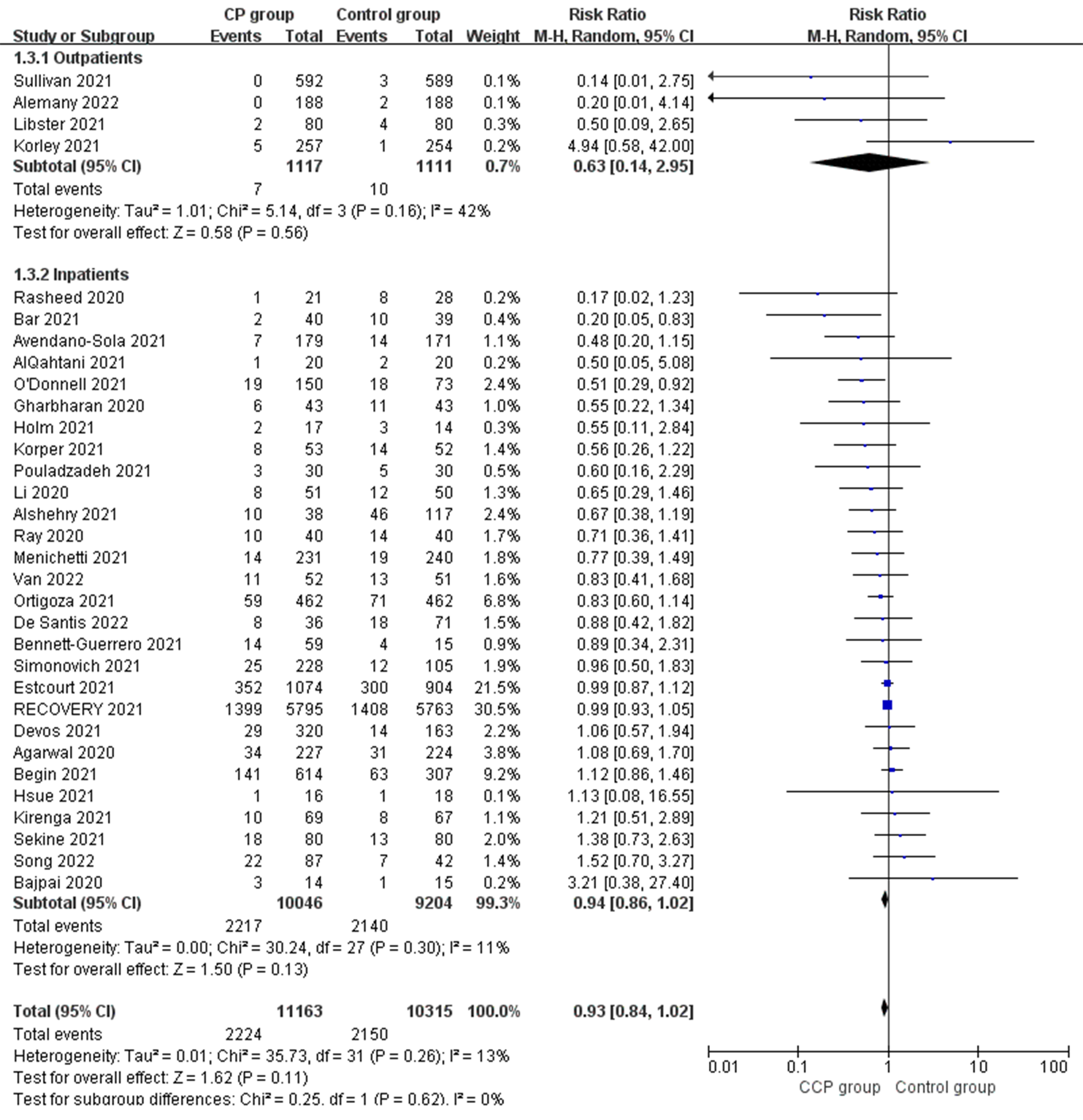


**Additional Figure 3:** Forrest plot of the risk ratio of 28-d mortality between CCP group and control group in the subgroups of outpatients and inpatients.


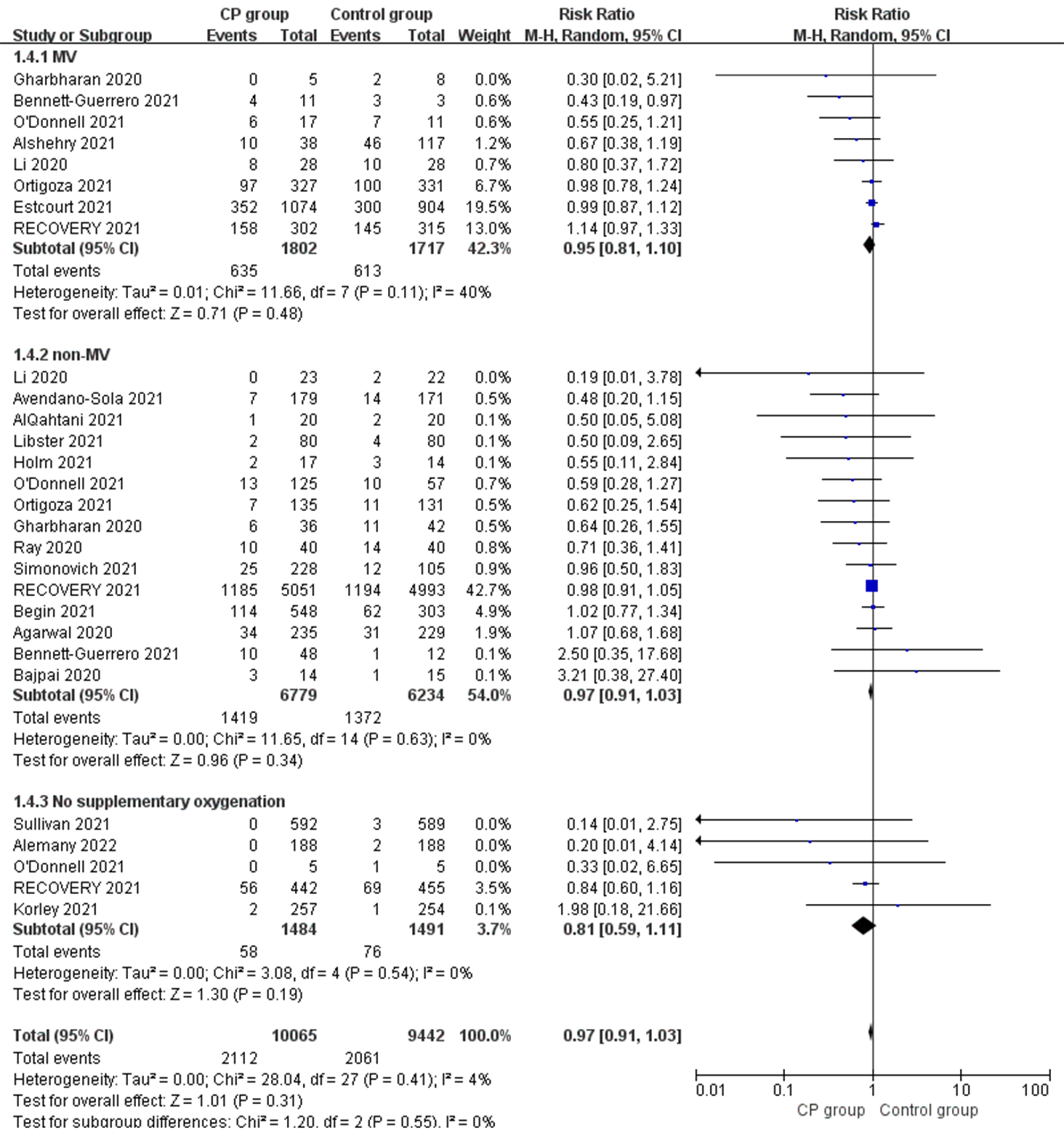


**Additional Figure 4:** Forrest plot of the risk ratio of 28-d mortality between CCP group and control group in the subgroups of patients requiring MV, non-invasive ventilation or no supplementary oxygenation at enrollment.


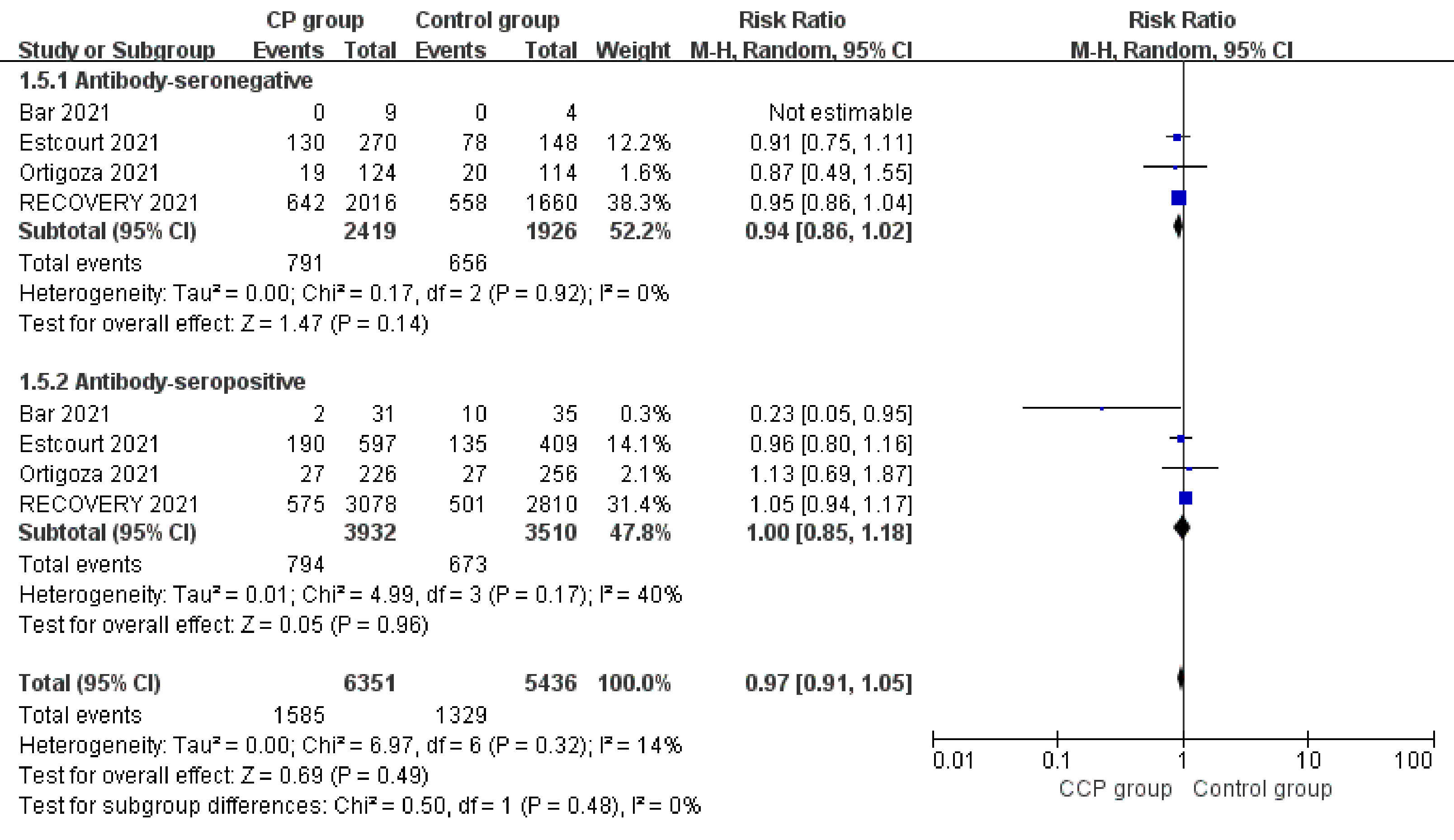


**Additional Figure 5:** Forrest plot of the risk ratio of 28-d mortality between CCP group and control group in the subgroups of antibody-seronegative and antibody-seropositive patients.


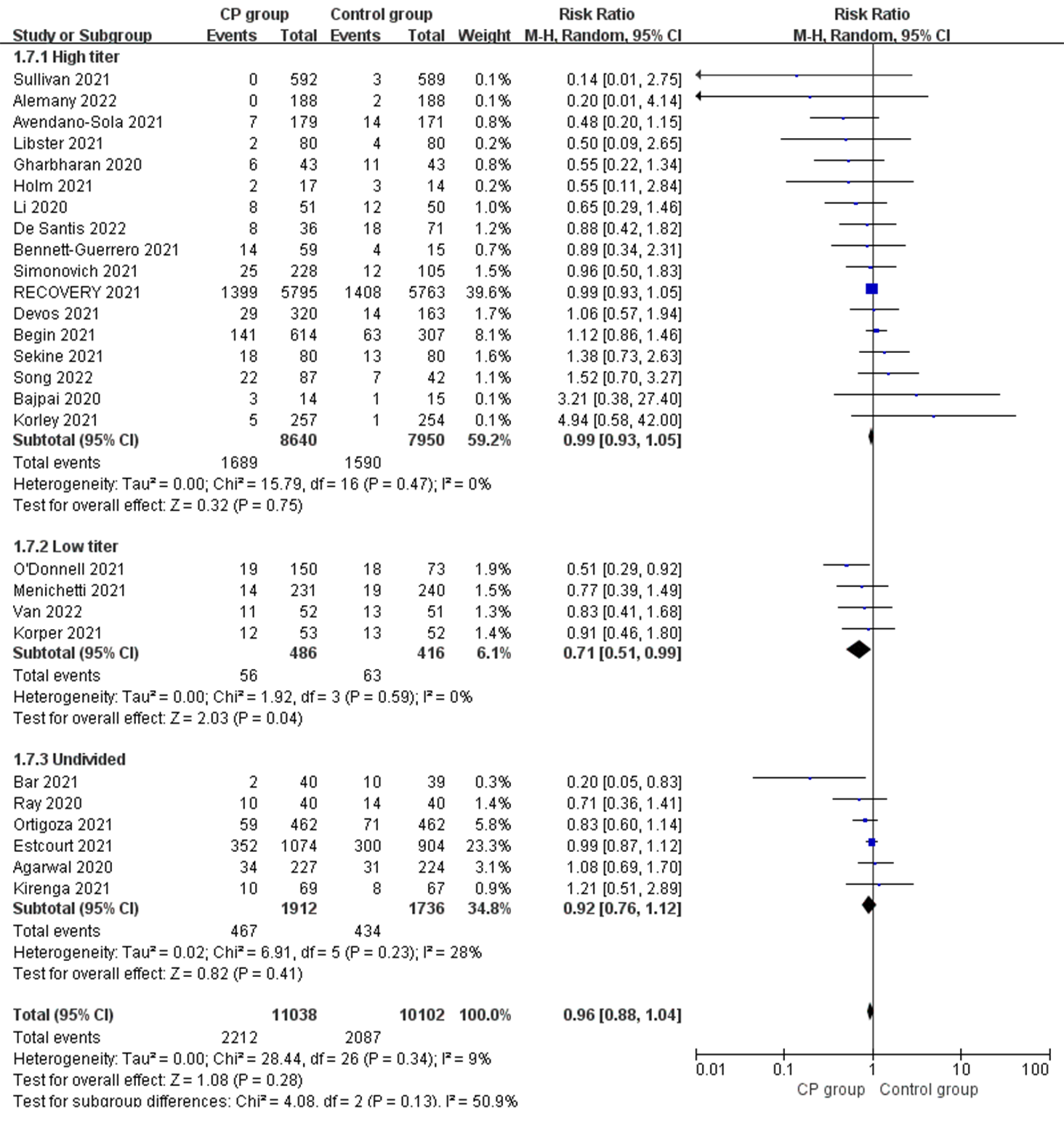


**Additional Figure 6:** Forrest plot of the risk ratio of 28-d mortality between CCP group and control group in the subgroups of patients receiving high titer CCP, low titer CCP or undivided titer of CCP


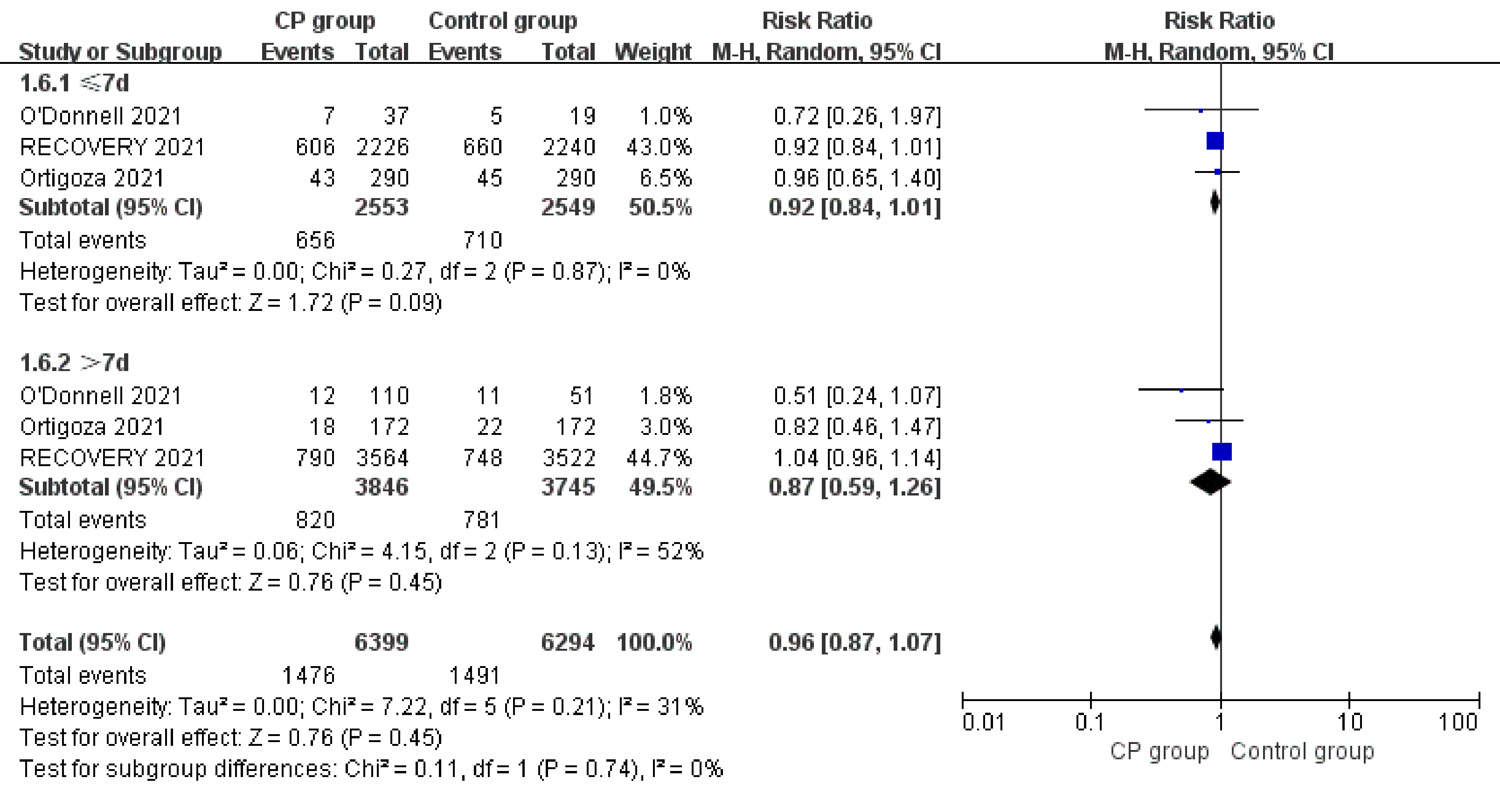


**Additional Figure 7:** Forrest plot of the risk ratio of 28-d mortality between CCP group and control group in the subgroups of patients with ≤7 days or ＞7 day from symptoms onset to enrollment.


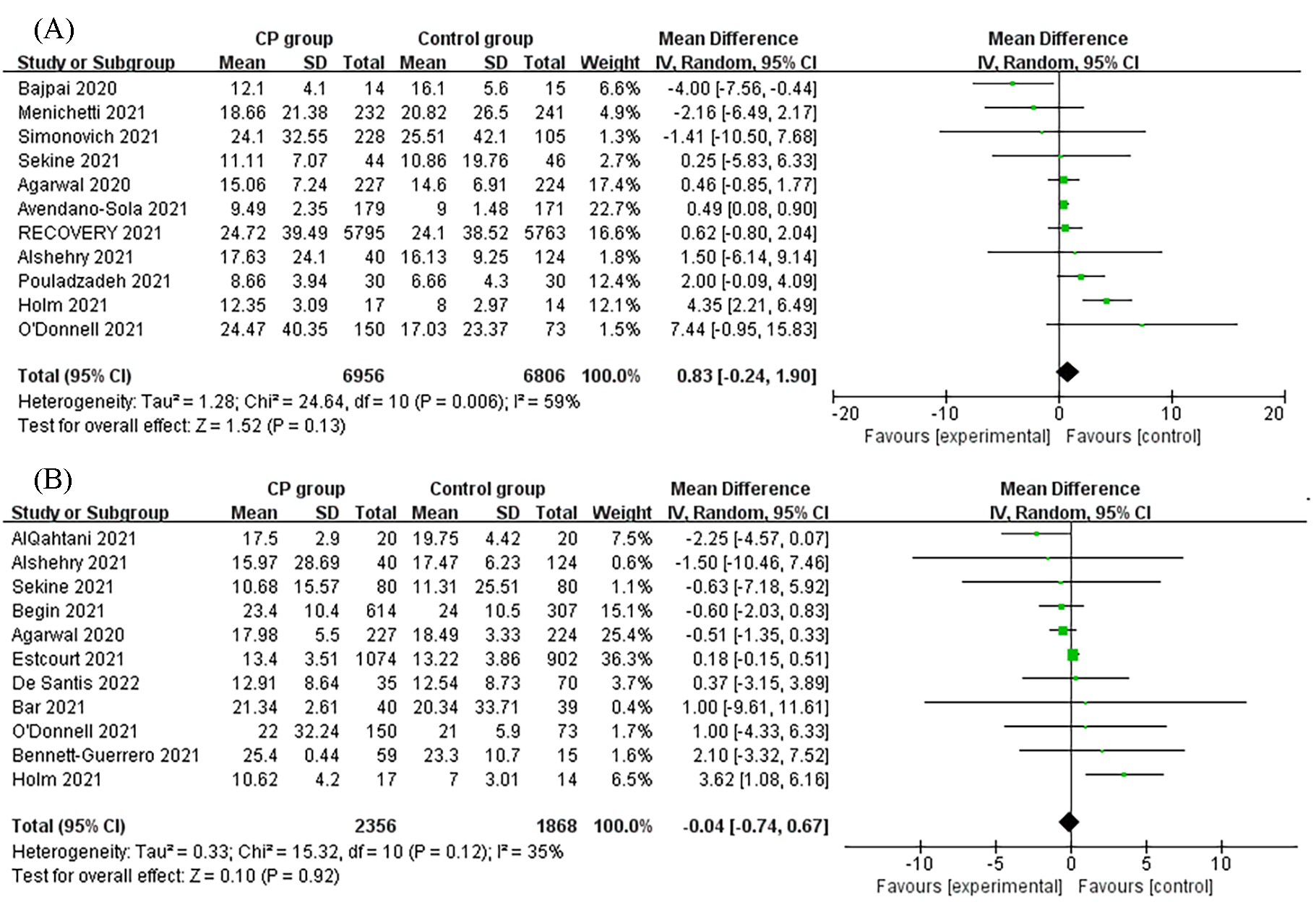


**Additional Figure 8:** Forrest plot of the mean difference of (A) Length of hospital stay and (B) Ventilation-free days between CCP group and control group.


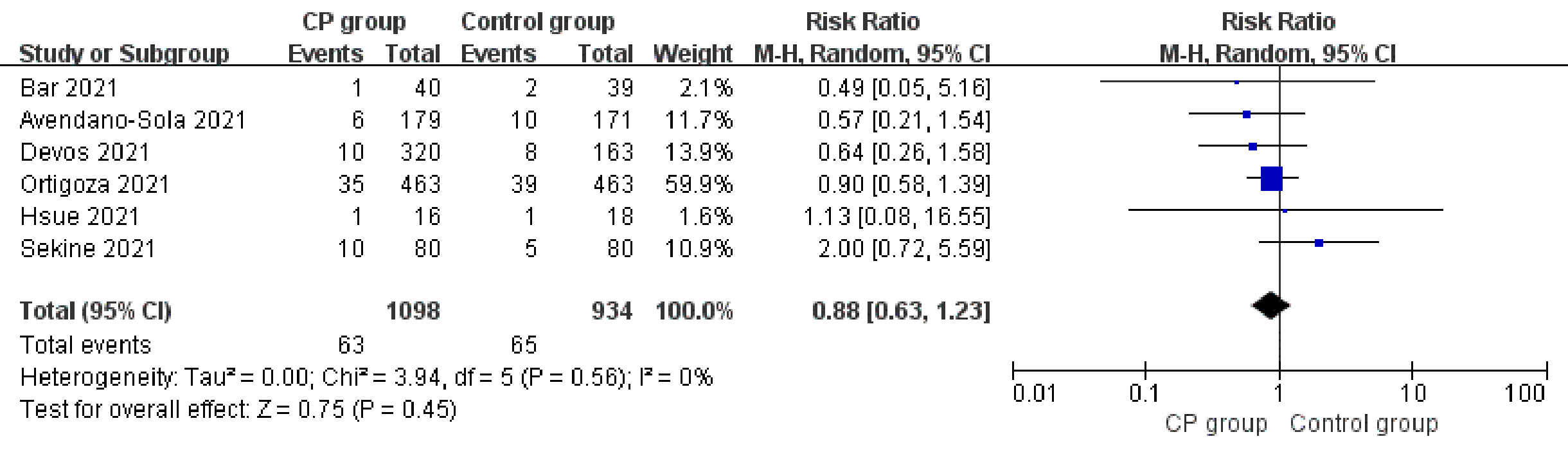


**Additional Figure 9:** Forrest plot of the risk ratio of 14-d mortality between CCP group and control group.


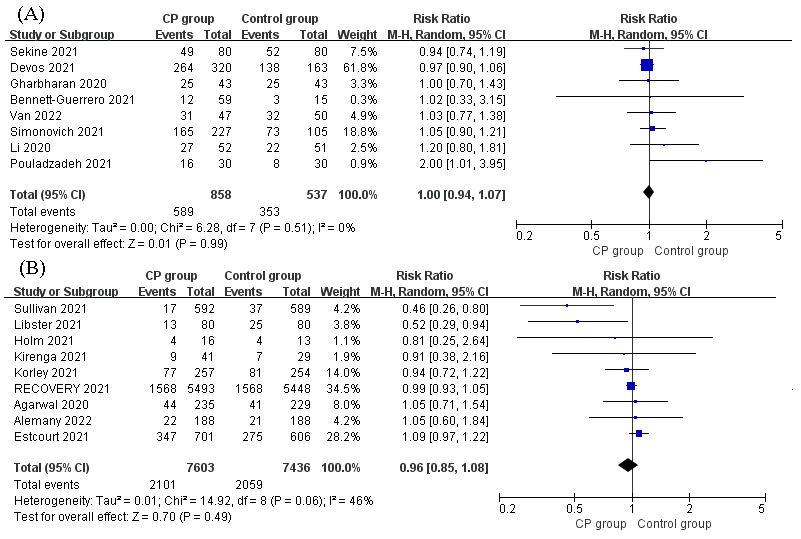


**Additional Figure 10:** Forrest plot of the risk ratio of (A) Improvements of symptoms and (B) progression of diseases between CCP group and control group.


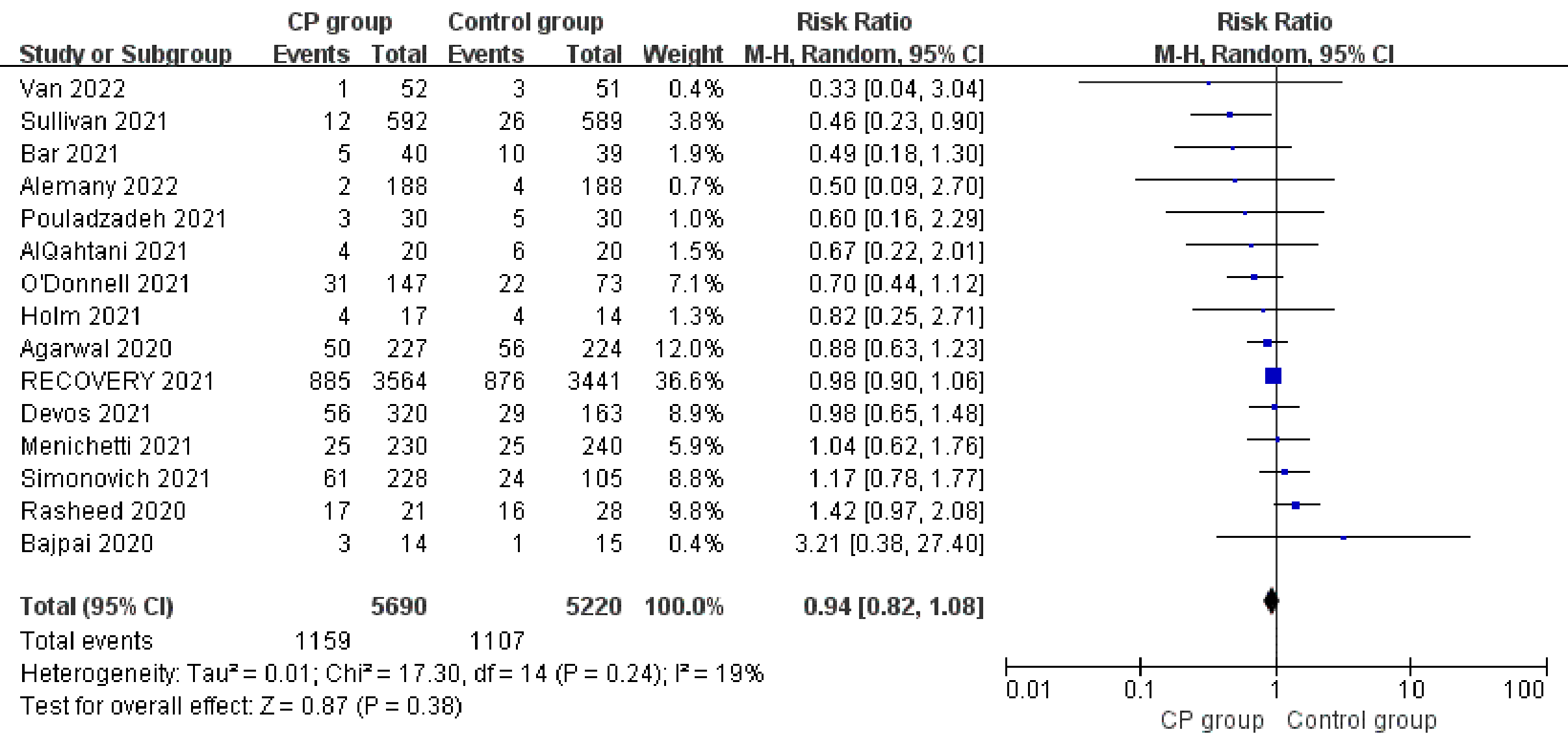


**Additional Figure 11:** Forrest plot of the risk ratio of requirement of mechanical ventilation between CCP group and control group.


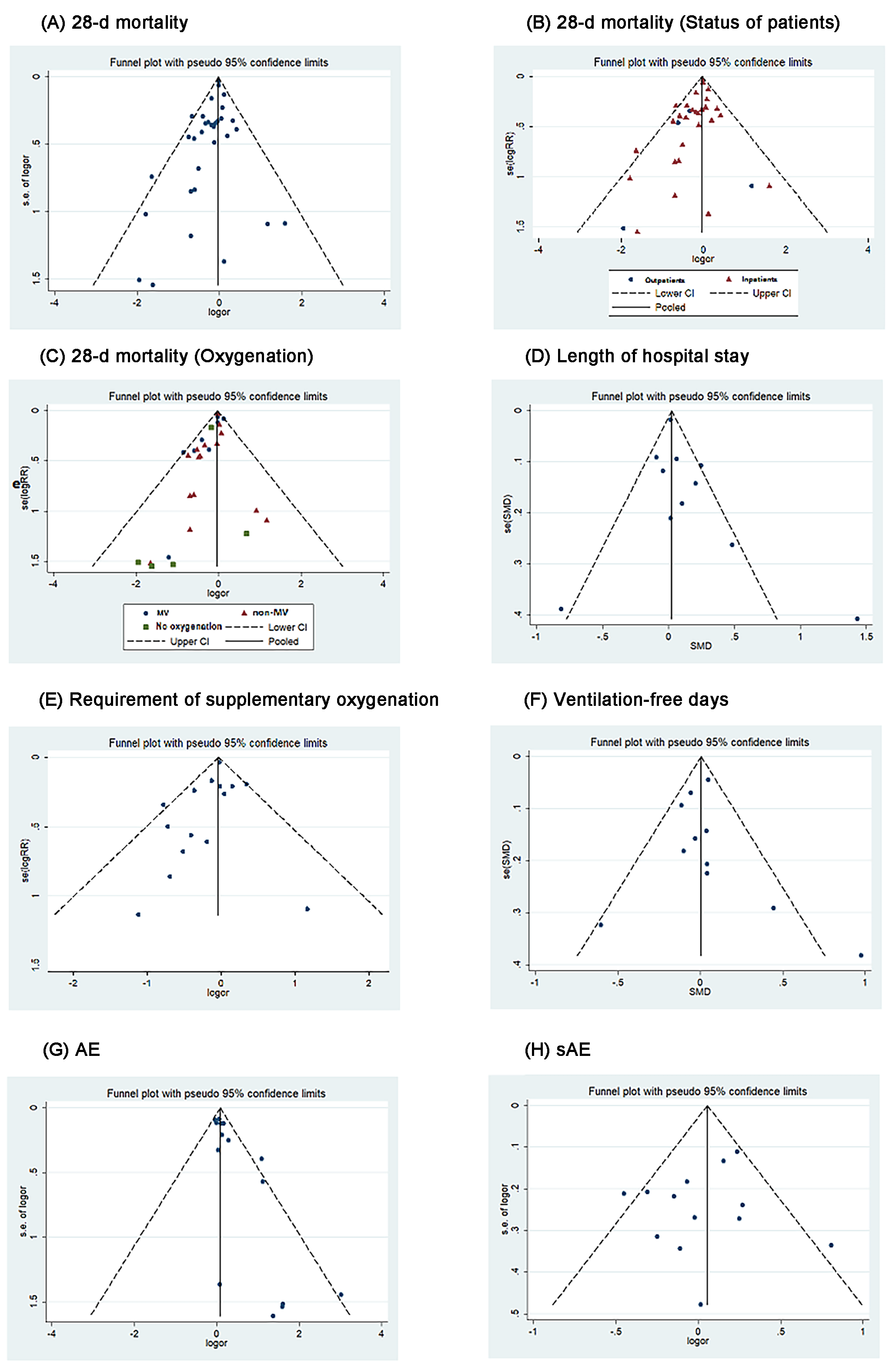


**Additional Figure 12**: Funnel plots of the effects estimates of the outcomes.
